# Lipopeptides from *Bacillus* Probiotics Can Target Transmembrane Receptors NOX4, EGFR, PDGFR, and OCTN2 Involved in Oxidative Stress and Oncogenesis

**DOI:** 10.3390/biotech15010004

**Published:** 2026-01-06

**Authors:** Evgeniya Prazdnova, Fadi Amirdzhanov, Anuj Ranjan, Radomir Skripnichenko

**Affiliations:** Academy of Biology and Medicine, Southern Federal University, Rostov-on-Don 344090, Russia; prazdnova@sfedu.ru (E.P.); randzhan@sfedu.ru (A.R.); radomirskr@inbox.ru (R.S.)

**Keywords:** probiotics, metabolites, carcinogenesis, molecular docking

## Abstract

*Bacillus*-derived lipopeptides are known to possess diverse biological activities, including antimicrobial and anticancer properties, though the mechanisms of such effects at the molecular level remain incompletely understood. We investigated whether non-ribosomal peptide metabolites from *Bacillus* can directly interact with transmembrane receptors implicated in oxidative stress regulation and cancer progression (NOX4, EGFR, PDGFR, and OCTN2) using molecular docking and 200 ns molecular dynamics simulations of 11 lipopeptide metabolites. Molecular docking revealed several strong ligand–protein interactions, with plipastatin and fengycin emerging as lead compounds demonstrating the highest binding affinities to multiple receptors. For NOX4, iturin D showed the strongest docking score of −7.85 kcal/mol. Fengycin demonstrated a high docking score of −7.38 kcal/mol for PDGFR and −8.1 kcal/mol for EGFR. Plipastatin showed the strongest docking scores of −11.12 kcal/mol for EGFR and −8.7 kcal/mol for OCTN2. Molecular dynamics simulations confirmed complex stability for these lead compounds, with protein RMSD remaining stable at ~1.5 Å and ligand RMSD between 1.9 and 6 Å over 200 ns. Our findings suggest that plipastatin and fengycin may act as modulators of key receptors involved in oxidative stress and cancer-related signaling. However, those *in silico* predictions require experimental validation. This work provides the first computational evidence of potential lipopeptide–receptor interactions and establishes a foundation for future experimental investigation of probiotic-derived therapeutics.

## 1. Introduction

The mechanisms of action in probiotics at the molecular level continue to be an active subject of exploration and study. The particular interest lies in the anticancer effects of probiotics, with increasing data in recent years and gaining the attention of the gut–cancer axis [1]. The bioactive metabolites of probiotic bacteria exhibit diverse biological activities beyond their well-established antimicrobial and antifungal properties, and can be considered as the agents of probiotics’ specific properties. Probiotic bacteria produce a variety of metabolites [2,3]; among these, non-ribosomal peptides (NRPs) possess several biological properties and potential as agents for complex intercellular interactions. NRPs are low-molecular-weight peptides and are characterized by their structural stability, including resistance to heat denaturation (up to 100 °C) and protease degradation, attributable to their incorporation of non-standard amino acids and stereoisomers [1,4].

Evidence suggests that the NRPs of probiotic strains exhibit not only antimicrobial and antifungal properties but also regulatory effects through activating or inactivating regulatory cascades [5]. For instance, bacillomycin activates apoptosis in gastric cancer cells [6], fengycin affects reactive oxygen species (ROS) generation and Ca^2+^ absorption [7], and surfactin contributes to the inhibition of several different types of cancer [8]. The catecholic siderophore bacillibactin is capable of forming complexes with iron ions [9], also exhibits antibacterial, antifungal, and anticancer activity [10,11]. Such evidence suggests that lipopeptide metabolites from the *Bacillus* species merit systematic investigation as potential modulators of key eukaryotic signaling receptors.

The transmembrane receptors examined in this study—NADPH-oxidase 4 (NOX4), Epidermal Growth Factor Receptor (EGFR), Platelet-Derived Growth Factor Receptor (PDGFR), and Organic Cation/Carnitine Transporter Novel 2 (OCTN2)—are interconnected in pathways governing oxidative stress regulation, cell proliferation, and drug transport; thus, influencing cancer progression. These receptors are reported to be involved in the complex pathway of oxidative stress and oncogenesis [12], share functional significance in cancer biology and are often targeted in treatment strategies. They were selected based on their connection in cancer pathways and treatment resistance. NOX4 has been shown to mediate resistance to EGFR-targeted therapies [13], while both EGFR and PDGFR are critical in signaling pathways that drive tumor growth and metastasis. OCTN2 may also influence how cancer cells respond to treatments by modulating drug transport [13,14].

NOX4 is a NADPH oxidase that catalyzes the process of production of ROS by reducing molecular oxygen, causes DNA damage, and leads to tumorigenesis [15,16]. It is also the only human NOX enzyme that can produce H_2_O_2_ relatively directly [17]. These ROS play a role in cell signaling and can influence tumor progression and resistance to therapies, particularly in cancers like non-small-cell lung cancer (NSCLC), where they are linked to EGFR-TKI resistance [13]. NOX4 is reportedly expressed in kidney cells and is a major producer of ROS [18]. In addition, normal renal function is restored after the inhibition of NOX [19], and either the inhibition of NOX4 or silencing of the gp91*^phox^* gene attenuates hHcys-induced inflammasome formation in podocytes [20]. NOX4 is also responsible for superoxide and hydrogen peroxide generation in hepatocytes during HCV (Hepatitis C virus) infection [21,22].

In cancer biology, NOX4 plays a dual role: while physiological ROS signaling supports normal cellular processes, pathological NOX4-driven ROS production contributes to genomic instability, tumor progression, and resistance to targeted therapies [23]. Notably, in non-small-cell lung cancer (NSCLC), NOX4 mediates resistance to EGFR-targeted tyrosine kinase inhibitor (TKI) therapy by enhancing IL-8 and PD-L1 expression, thus promoting immune evasion and therapeutic resistance [13].

Activation of tyrosine kinase receptors of EGFR and PDGFR may also lead to oxidative stress because they tend to enhance cell growth and proliferation [24]. EGFR (Epidermal Growth Factor Receptor) is a receptor tyrosine kinase that promotes cell proliferation and survival. Its dysregulation is a common feature in various cancers, where activating mutations drive oncogenic signaling through the PI3K/AKT and MAPK/ERK pathways [13].

PDGFR (Platelet-Derived Growth Factor Receptor) is another receptor tyrosine kinase that regulates cell growth and division and is implicated in the pathology of several tumors [13]. PDGFR dysregulation contributes to multiple malignancies, including gastrointestinal stromal tumors (GIST), chronic myeloid leukemia (with BCR-ABL fusion activating PDGFR-associated pathways), and various solid tumors. There is evidence that PDGFR may play an important role in the proliferation of breast cancer cells [25]. Resistance to PDGFR-targeted therapies develops through kinase domain mutations and the engagement of compensatory signaling pathways [26,27].

OCTN2 (Organic Cation/Carnitine Transporter Novel 2) is a transporter that affects the uptake of various compounds, including drugs, influencing cellular responses and potentially impacting cancer treatment outcomes [14]. It is involved in oxidative stress regulation and DNA repair [28], and plays a complex and context-dependent dual role in cancer biology.

OCTN2 is a high-affinity, sodium-dependent transporter with primary substrate specificity for L-carnitine (Km = 4.34 μM). In many cancer types, OCTN2 expression is upregulated and exerts tumor-promoting effects by facilitating carnitine uptake, which fuels mitochondrial fatty acid β-oxidation and provides alternative energy sources [29,30]. But beyond its carnitine transporter role, OCTN2 functions as a polyspecific organic cation transporter with broad substrate specificity for numerous chemotherapeutic agents, including oxaliplatin, etoposide, imatinib, and verapamil. This latter property can exert tumor-suppressive effects: in esophageal squamous cell carcinoma (SCC), patients with high OCTN2-expressing tumors receiving oxaliplatin-based chemotherapy demonstrate significantly better therapeutic response, reduced recurrence rates, and prolonged overall survival compared to patients with low OCTN2 expression. This context reflects OCTN2-mediated drug accumulation, enhancing intracellular chemotherapy concentration and cytotoxic efficacy [31].

Thus, OCTN2 represents a pharmacologically complex target with opposing functions depending on context: inhibiting OCTN2 might suppress carnitine-dependent metabolic transformation and proliferation in tumors where it functions as a growth promoter, while enhancing or selectively activating OCTN2 might facilitate chemotherapy uptake and efficacy in other tumor contexts.

Considering this, whether target lipopeptides bind OCTN2 to facilitate or impede carnitine transport, or whether they serve as OCTN2 substrates, will fundamentally determine the net biological consequence of this binding—tumor-suppressive or tumor-promoting.

Protein–protein interaction network analysis confirms the functional clustering of these four proteins, with NOX4 acting as a regulatory hub influencing EGFR/PDGFR signaling and immunomodulatory gene expression [13].

Each of our selected target proteins also represents an established or emerging therapeutic target: selective NOX4 inhibitors (GKT137831/Setanaxib) are in clinical development [32]; EGFR and PDGFR inhibitors are FDA-approved for multiple cancers [33]; and OCTN2 is increasingly recognized as a target for enhancing drug delivery and regulating cancer cell metabolism [34]. Overall, the dysregulation of these proteins plays a critical role in the pathogenesis and progression of tumors [12,18]. Computational approaches, particularly molecular docking and dynamics simulations, present a powerful and rational strategy for the initial screening and mechanistic deconvolution of potential drug-receptor interactions [35]. In the context of probiotic metabolites, which often constitute complex mixtures of structurally diverse compounds, *in silico* methodologies provide an indispensable framework for prioritizing the most promising candidates for subsequent costly and time-consuming experimental validation [36]. Specifically, this paradigm allows researchers to do the following: (1) rapidly evaluate binding energy and polypharmacology across multiple therapeutic targets; (2) identify key molecular interactions, such as hydrogen bonding and hydrophobic contacts, that govern ligand-receptor recognition [37]; and (3) generate robust, testable hypotheses concerning structure-activity relationships. For this study, computational modeling serves as an essential first step in bridging the observed biological effects of *Bacillus* probiotics with their putative molecular mechanisms of action, thereby guiding a more targeted and efficient experimental design for validating these interactions *in vitro* and *in vivo* [38].

The goal of this work was to determine whether non-ribosomal metabolites produced by *Bacillus* probiotics can target NOX4, EGFR, PDGFR, and OCTN2 through molecular docking and dynamics simulations, and to generate testable hypotheses regarding potential mechanisms by which probiotic lipopeptides exert their reported anticancer and antioxidant effects.

## 2. Materials and Methods

### 2.1. Preparation of Protein Structures

The PDB structures of four membrane receptors—NOX4 (AlphaFoldDB Q9NPH5), EGFR (PDB ID 1M17), PDGFR (PDB ID 5GRN), and OCTN2 (AlphaFoldDB O76082)—were imported into Discovery Studio Visualizer (v4.0, BIOVIA, Dassault Systèmes, France), and co-crystal ligands and other heteroatoms were removed. The AlphaFoldDB structures were already clean. They were checked for quality, and polar hydrogens were added and stored as .pdb files for further use. Further optimization was performed using an energy minimization program using 100 cycles of each steepest descent and conjugate gradient in Chimera (v. 1.6.2, University of California, San Francisco, CA, USA). The resulting .pdb files were verified using the PROCHECK server “https://saves.mbi.ucla.edu/” (accessed on 13 September 2024) and aligned with the parent structure to rule out any visual anomaly in the structure.

### 2.2. Preparation of Ligand Structures

Ligand molecules were nine small peptides, namely, bacillibactin (CID 125349), bacilliomycin (CID 3086051), fengycin (CID 443591), fusaracidin A (CID 21581469), fusaracidin B (CID 139583239), iturin C (CID 3085004), iturin D (CID 158570), mycosubtilin (CID 3083700), plipastatin (CID 5491283), polymixin B (SID 381745019), and surfactin (CID 443592). For comparative analysis, reference inhibitors were also prepared: gefitinib (CID 123631), erlotinib (CID 176870), imatinib (CID 5291), carnitine (CID 288), and the selective NOX4 inhibitor GLX351322 (CID 2697686). The ligands were prepared using PerkinElmer ChemOffice software by obtaining the chemical structures of the compounds in .sdf file format in ChemDraw (v. 16.0, PerkinElmer Informatics, Waltham, MA, USA), their geometry optimization in Chem3D (v. 16.0, PerkinElmer Informatics, Waltham, MA, USA) using the MM2 force field, and Gasteiger charges featurization [39]. Ligands were further imported into the AutoDockTools (v. 1.5.7, Molecular Graphics Laboratory, The Scripps Research Institute, La Jolla, CA, USA) H-atoms were added, and they were stored in the .pdbqt format.

### 2.3. Molecular Docking

Molecular docking with Q9NPH5, 1M17, 5GRN, and O76082 was carried out using AutoDock Vina 4.0 [40]. Following the preparation steps described above, the optimized receptor and ligand .pdbqt files were imported into AutoDockTools 1.5.7 for grid-box generation. Binding sites for each receptor were selected based on curated annotations from the UniProt database. For Q9NPH5, the grid was centered on the catalytic ferric oxidoreductase domain (residues 58–303), incorporating the E-loop (218–273) and the FAD-binding domain (304–419) [41,42]. The binding site for 1M17 was defined as its canonical ATP-binding pocket, encompassing the nucleotide-binding loop (718–726) [43,44]. Similarly, for 5GRN, the ATP-binding site was targeted, including the glycine-rich P-loop (599–607) and the catalytic aspartate (Asp818) [45,46], while the docking site for O76082 was based on a putative substrate-binding region, specifically the conserved motif (residues 218–225) [47,48]. Using the graphical interface of AutoDock 4.2 MGL Tools 1.5.6 [49], grid boxes were prepared to encapsulate these defined binding sites in their entirety, with the following parameters: for Q9NPH5, center x = −10.861, y = −5.500, z = −3.139, dimensions 84 × 68 × 76 Å; for 1M17, center x = 28.592, y = −2.430, z = 44.665, dimensions 48 × 48 × 62 Å; for 5GRN, center x = 18.250, y = −11.056, z = −11.417, dimensions 30 × 40 × 40 Å; and for O76082, center x = −7.778, y = −6.111, z = 2.500, dimensions 40 × 40 × 40 Å. The vina was run through a command line having a grid point spacing of 0.375 Å in configuration with an exhaustiveness value of 8. The output data was analyzed using the Discovery Studio visualizer [50].

Docking validation was performed using standard internal procedures. For receptors with co-crystallized ligands (EGFR and PDGFR), the native ligand was re-docked to confirm recovery of the experimental binding mode within the accepted RMSD threshold (<2 Å). 

### 2.4. Molecular Dynamics Simulation

Using the Desmond v7.6 utility from the Schrödinger suite [51], a molecular dynamics simulation was conducted to assess the stability of the ligand–protein complex of the lead compounds (iturin C, iturin D, and plipastatin) with the Q9NPH5 over a period of 200 ns at 300 K and 1.01325 bar (NPT ensemble) using the OPLS4 force field and the TIP3P water model [51]. The systems were prepared using the System Builder tool. An orthorhombic simulation box was defined with a 10-Å buffer distance from the protein surface in each direction. Systems were neutralized with Na^+^ counterions, and a physiological concentration of 0.15 M NaCl was added [51].

Although crystallographic heteroatoms were removed prior to docking, all ligand–protein complexes were subsequently subjected to 200 ns MD simulations under explicit solvation (TIP3P model). This dynamic environment enables the formation and stabilization of water-mediated hydrogen bonds and other heteroatom-assisted interactions that may not be captured in the initial docking poses. Thus, potential water-bridge contributions to ligand binding were evaluated during MD rather than relying solely on static docking structures.

The system was neutralized with Na^+^ counterions. For protein preparation and energy minimization, the built-in Desmond utility was used with default parameters: a convergence threshold of 1.0 kcal/mol/Å and a maximum of 2000 iterations. MD trajectory analysis, including RMSD of protein Cα atoms, ligand RMSD, and protein–ligand interaction profiles, was carried out using the Simulation Event Analysis and Simulation Interaction Diagram (SID) tools included in the Desmond Molecular Dynamics System version 7.6 within Schrödinger Suite 2023-4 [51].

### 2.5. PPI Network Analysis

Protein–protein interaction (PPI) analysis was performed using the STRING database (https://string-db.org/) to contextualize the biological relevance of the four selected receptors (NOX4, EGFR, PDGFR, and OCTN2) within cellular signaling pathways. The PPI networks for NOX4, EGFR, PDGFR, and OCTN2 were generated using the STRING database (confidence score ≥ 0.4). Network parameters, including degree and betweenness centrality, were used to assess connectivity and functional relevance. Functional enrichment provided by STRING was used to identify shared biological processes and signaling pathways associated with the selected receptors. The PPI analysis served to contextualize the four pre-selected targets within oxidative stress and membrane-associated signaling networks.

## 3. Results

### 3.1. Docking with NADPH Oxidase 4

After the docking with Q9NPH5 in the defined binding site of residues 15–105 [52], the compound iturin D was ranked first with a binding energy of −7.85 ± 0.05 (RMSD 1.193 Å LB and 1.153 Å UB) and ΔG of −17.996 kcal/mol. The plipastatin was ranked second with −7.75 ± 0.03 kcal/mol (RMSD 1.195 Å LB and 2.308 Å UB) and ΔG of −23.20 kcal/mol. However, the third rank compound was iturin C with a binding energy of −7.65 ± 0.06 kcal/mol (RMSD 2.696 Å LB and 2.599 Å UB) and ΔG of −18.70 kcal/mol.

**Figure 1:**
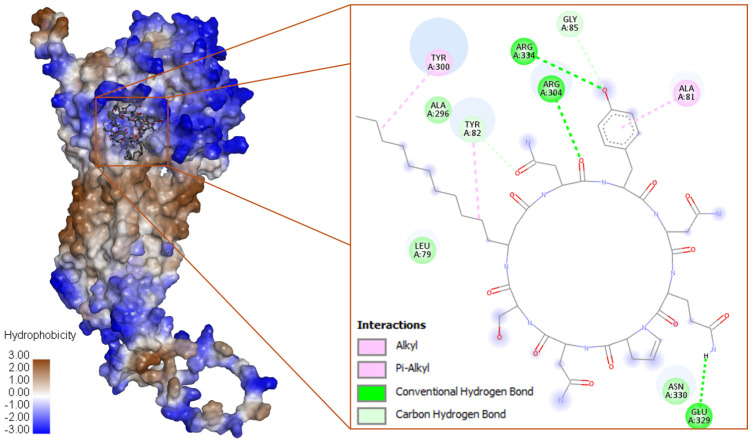
Space–filled model of Q9NPH5 with top poses of iturin D, the binding cavity has been highlighted for better clarity, followed by a 2D and 3D representation of the interaction map with binding site residues.

**Figure 2:**
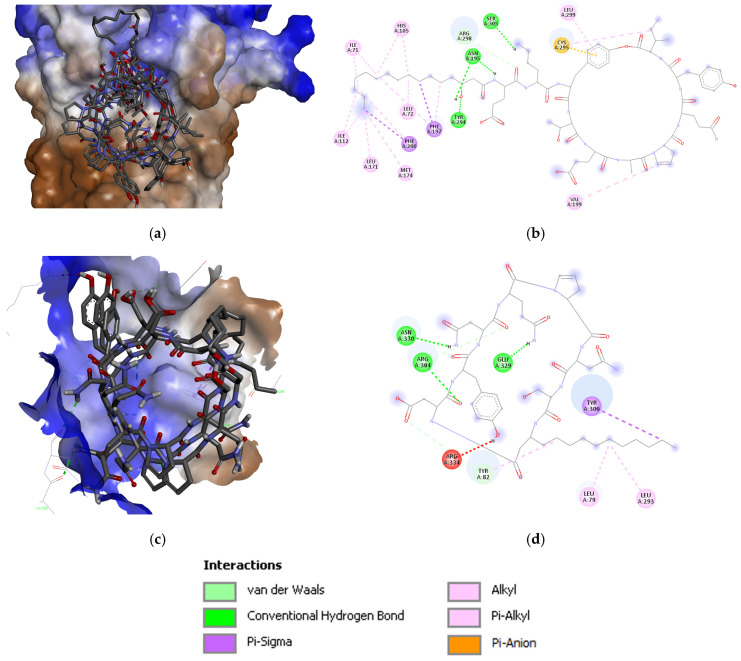
Plipastatin (**a**) 3D and (**b**) 2D interaction maps; (**c**) iturin C (bottom) 3D and (**d**) 2D interaction maps with Q9NPH5.

**Figure 3:**
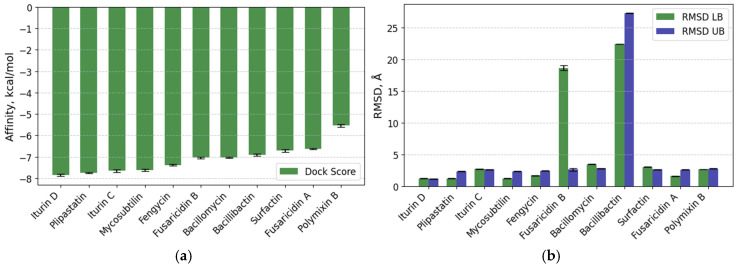
(**a**) The binding energy (kcal/mol) of each nonribosomal peptide with the Q9NPH5 receptor is presented; (**b**) information about the RMSD of ligands (Å) is provided, where RMSD L.B. represents the lower bound of RMSD, and RMSD U.B. represents the upper bound of RMSD.

The iturin D interacts with three conventional and two carbon H-bonds (Hydrogen bonds) conferred by Arg304, Glu329, Arg334, and Tyr82; and Gly85, respectively (Figure 1). Three π-alkyl bonds were contributed by Ala81, Tyr82, and Tyr300. The hydrophobic alkyl chain is significantly stabilized by Tyr82 and Tyr300 present in the water accessible area of the cavity. The plipastatin binds with Q9NPH5 in the binding pocket by four conventional H bonds contributed by Asn195, Tyr294, and Ser305; however, one carbon H-bond was contributed by Arg298. Aromatic amino acids, His105, Phe197, and Phe200, are actively involved in establishing the π-σ and π-alkyl interactions (Figure 2). Cys295 establishes a π-sulfur interaction, and it is also placed in the water accessible area for electrostatic interaction. Iturin C (Figure 2) had a similar interaction pattern as iturin D, with conventional H-bonds conferred by Arg304, Glu329, and Asn330 and two carbon H-bonds by Tyr82 and Arg304. A π-σ and π-alkyl is contributed by Tyr300 and Tyr82. The results of docking NRP with the active center of Q9NPH5 are shown in Figure 3. Detailed binding energy data for the investigated lipopeptides interacting with the receptor Q9NPH5 are provided in the Supplementary Materials (Tables S1–S3).

### 3.2. Docking with Epidermal Growth Factor Receptor Tyrosine

The plipastatin exhibited excellent dock score binding energy with EGFR (PDB ID 1M17), which was −11.12 ± 0.5 kcal/mol (RMSD 1.987 ± 0.04 LB and 1.678 ± 0.05 UB), and a ΔG of −21.34 kcal/mol, followed by fengycin (binding energy −9.76 ± 0.5 kcal/mol with RMSD 2.345 ± 0.05 LB and 2.596 ± 0.05 UB; and ΔG of −21.97 kcal/mol). The third-ranked compound was polymixin B with the binding energy of −7.92 ± 0.06 kcal/mol (RMSD 1.825 ± 0.04 LB and 1.596 ± 0.04 UB) and ΔG of −19.87 kcal/mol.

**Figure 4:**
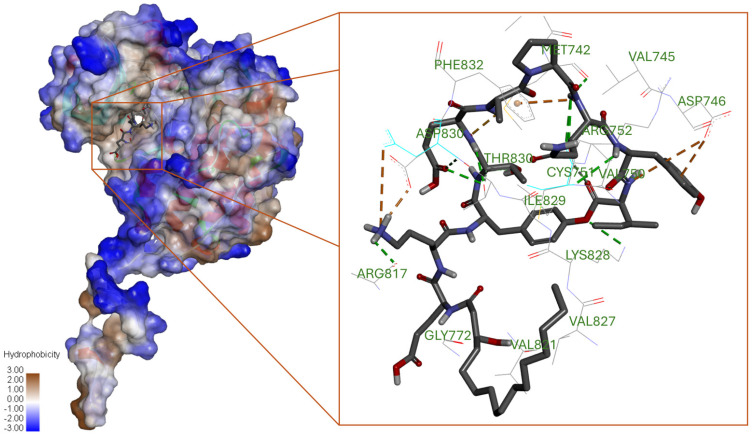
Space–filled model of 1M17 interacting with plipastatin, illustrated with binding cavity and 3D pattern of interaction with binding site residues.

The plipastatin interacted with nine conventional H-bonds and two carbon-H bonds contributed by Met742, Cys751, Arg817, Lys828, Thr830, and Asp831; and Met742, Asp746, and Phe832, respectively (Figure 4). A π-cation was contributed by the phenyl ring of Phe832, a π-anion by Asp746, and a sulfur bond by Met742.

**Figure 5:**
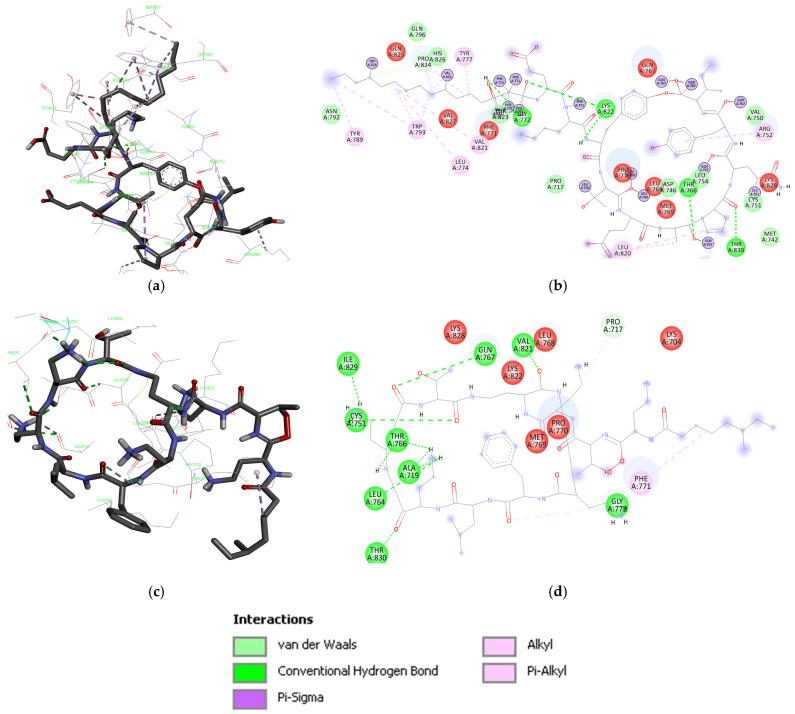
Fengycin (**a**,**b**) and polymyxin B (**c**,**d**) in the binding cavity of 1M17 and 2D mapping of interacting residues.

Fengycin interacts with 1M17 by five conventional H-bonds contributed by Gly772, Thr766, Lys822, and Thr830 (Figure 5). However, the third-ranked compound polymyxin B interacted with 1M17 by nine conventional H-bonds contributed by Ala719, Cys751, Leu764, Thr766, GLN767, and ILE829, and two conventional H-bonds contributed by Gly772 and Pro717. A π-alkyl bond was also observed shared by Phe771 (Figure 5). Detailed binding energy data for the investigated lipopeptides interacting with the receptor 1M17 are provided in the Supplementary Materials (Tables S5–S8).

### 3.3. Docking with Platelet-Derived Growth Factor Receptor

Iturin D, iturin C, and fusaricidin B were the top three ranked compounds after docking with PDGFR (PDB ID 5GRN). The iturin D had a dock binding energy of −8.76 ± 0.09 kcal/mol (RMSD 2.206 ± 0.03 LB and 3.373 ± 0.07 UB) with a ΔG of −21.11 kcal/mol, followed by iturin C with a dock binding energy of −8.56 ± 0.05 kcal/mol (RMSD 2.224 ± 0.05 LB and 3.474 ± 0.07 UB) and a ΔG of −19.49 kcal/mol. The third compound was fusaricidin B with a dock binding energy of −8.34 ± 0.05 kcal/mol (RMSD 1.133 ± 0.03 LB and 1.272 ± 0.04 UB) and a ΔG of −20.63 kcal/mol.

**Figure 6:**
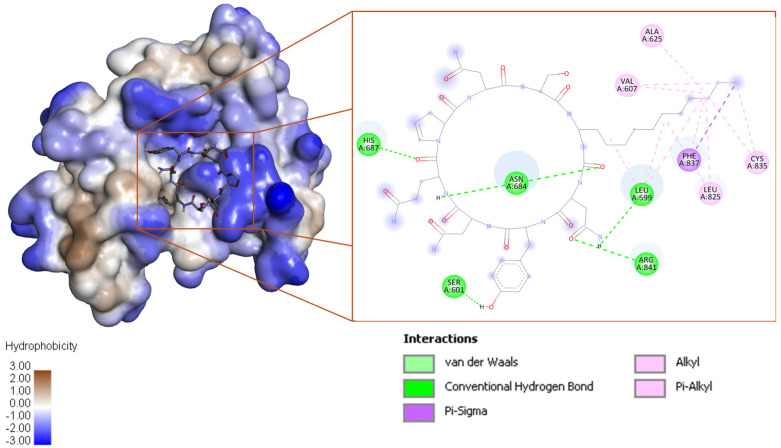
Iturin D in the binding cavity of the space–filled model of 5GRN with 3D and 2D mapping of interacting residues.

The iturin D interacts with 5GRN by six conventional H-bonds contributed by Leu599, Ser601, Asn684, His687, and Arg841, and one each of π-σ and π-alkyl bonds supported by Phe837 (Figure 6). Fusaricidin interacts with eight conventional H-bonds and one carbon H-bonds contributed by Ser643, Val815, His816, Asp818, and Asp836; and Asp836, respectively. A π-alkyl bond was observed to be supported by His816. The third-ranked compound fengycin interacts with 5GRN by six conventional H-bonds, one carbon H-bond, and one π-donor H-bond. The conventional H-bonds were contributed by Arg597, Val598, Asp681, and Asn848. The carbon H-bonds and π-donor H-bonds were supported by Ser847. Detailed binding energy data for the investigated lipopeptides interacting with the receptor 5GRN are provided in the Supplementary Materials (Tables S9–S12).

### 3.4. Docking with Organic Cation/Carnitine Transporter-2

Bacillomycin, plipastatin, and fengycin were found to be the three top-ranked compounds after docking with OCTN2 (AlphaFoldDB O76082). Their dock binding energies were −9.66 ± 0.05 kcal/mol (RMSD 2.329 ± 0.06 LB and 2.655 ± 0.05 UB), −8.7 ± 0.05 kcal/mol (RMSD 2.233 ± 0.04 LB and 3.755 ± 0.07 UB), and −6.75 ± 0.04 kcal/mol (RMSD 1.943 ± 0.04 LB and 2.4952 ± 0.05 UB), respectively. The free-energy ΔGs were −21.36, −27.95, and −20.61 kcal/mol, respectively.

**Figure 7:**
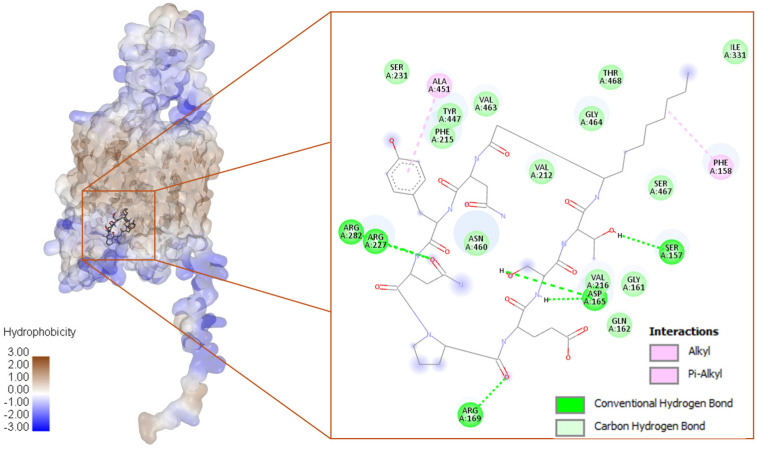
**Left**: Bacillomycin in the binding cavity of a space–filled model of O76082 (reduced transparency), along with 2D and 3D mapping of interacting residues. **Right**: Bacillomycin is docked at the bottom of the gorge formed by the helices, locking the visibility; hence, the ribbon model was used to illustrate the bacillomycin binding.

The bacillomycin interacts with nine conventional H-bonds contributed by Ser157, Asp165, Arg169, Arg227, and Arg282 (Figure 7). Two π-alkyl interactions were observed to be supported by Phe158 and Ala451.

**Figure 8:**
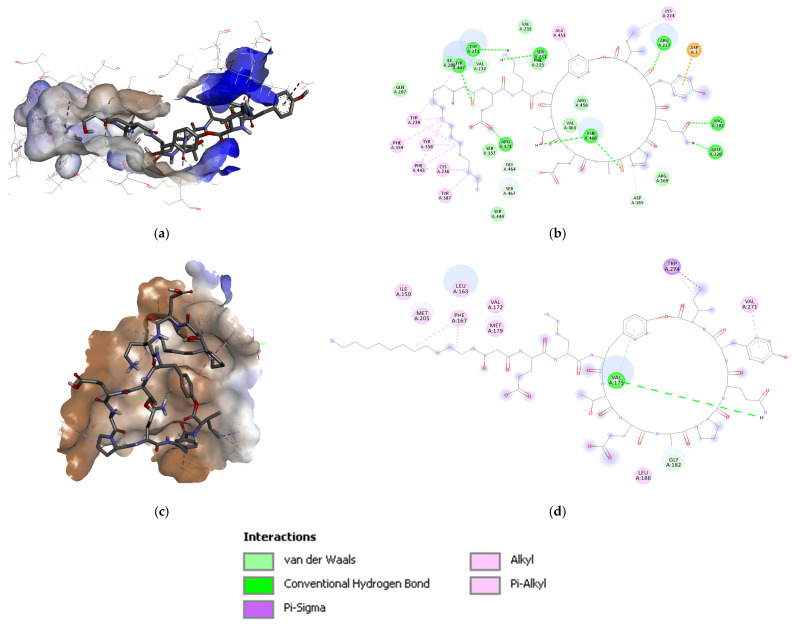
(**a**) Plipastatin and (**c**) fengycin binding in the binding cavity of O76082 (**a**,**c**), and (**b**,**d**) 2D mapping of interacting residues.

The plipastatin interacts with O76082 with eleven conventional H-bonds, five carbon H-bonds, and one π-anion interaction (Figure 8). The conventional H-bonds were contributed by Tyr211, Glu220, Arg227, Ser231, Arg282, Tyr447, Asn460, and Arg47. The carbon H-bonds were contributed by Arg282, Asn460, Gly464, and Ser467. One π-anion was supported by Asp165.

The third-ranked compound fengycin interacted with one conventional H-bond contributed by Val175 and two carbon H-bonds supported by Gly182 (Figure 8). Apart from H-bonds, five π-alkyl interactions were observed to be supported by Phe167, Val175, Val271, and Trp274. One π-sigma interaction was also noticed, which was supported by Trp274. Detailed binding energy data for the investigated lipopeptides interacting with the receptor O76082 are provided in the Supplementary Materials (Tables S13–S17).

### 3.5. MD Simulations

**Figure 9:**
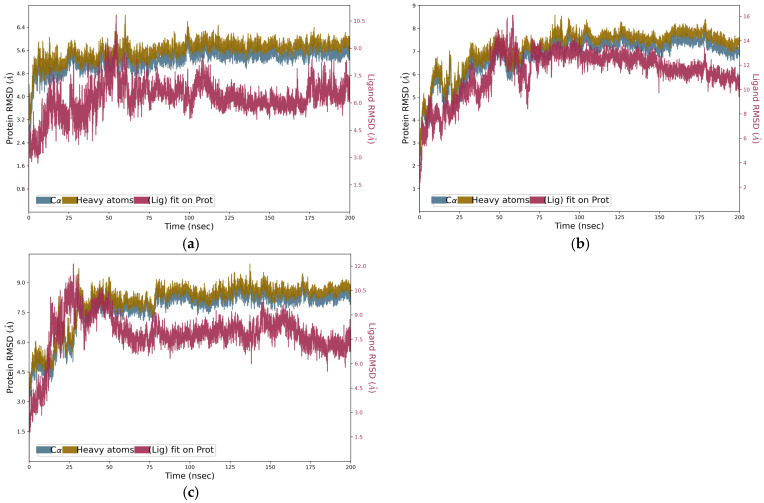
Protein–ligand RMSD plot for 200 nsec depicting complex stability from approximately 70 nsec to 200 nsec for (**a**) iturin D, (**b**) plipastatin, and (**c**) iturin C with Q9NPH5.

The RMSD graph was observed to analyze the Cα atoms of the protein and was found to be stable and converged with an average deviation observed of 1.5 Å from the beginning to the end of the 200 ns simulation (Figure 9). In all cases, the formation of the strongest ligand–protein complexes was observed around 50 nsec, and this stability was maintained until 150 nsec for the NOX4–iturin D complex. The molecular dynamics trajectories revealed stable behavior of the protein backbone, with the Cα RMSD converging at approximately 1.5 Å for all complexes (Figure 9). The ligand RMSD values exhibited greater flexibility, ranging between 1.9 and 6.0 Å, indicative of conformational mobility and adjustments within the binding pocket. Crucially, despite this flexibility, the core binding mode predicted by docking remained engaged. Analysis of the interaction timelines (Supplementary Figure S2) and per-residue contact occupancy (Supplementary Figure S1) confirms that the key hydrogen bonds and hydrophobic interactions identified in the docking poses—such as those involving Arg304 and Tyr82 for iturin D/NOX4—were maintained for a significant fraction (>50–70%) of the simulation time. This persistent interaction network, rather than absolute ligand rigidity, underpins the observed stability of the complexes. The protein and ligand RMSF plots and the temporal representation of the receptor–ligand interactions, including detailed characterization of molecular contacts (H-bonds, hydrophobic interactions, ionic bonds, and water bridges), are presented in the Supplementary Materials (Figures S1–S4).

### 3.6. STRING Analysis of Target Receptors

The targets selected for the docking of NRP molecules, as it was described previously, hold varied significant importance in terms of molecular signaling and consequences leading to multiple diseases, such as diseases of cellular proliferation and intestinal diseases. Their co-expression regulates multiple pathways simultaneously, possibly leading to the expression in several cell lines.

**Figure 10:**
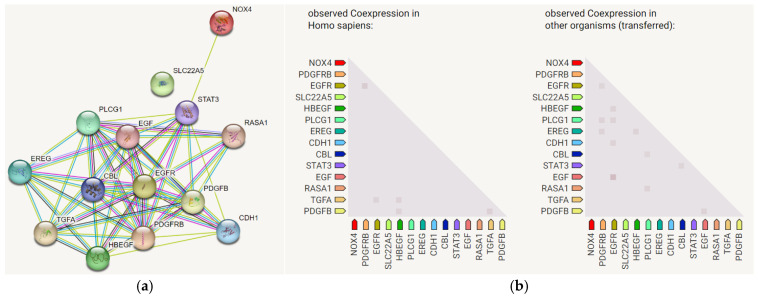
(**a**) STRING analysis of NOX4, EGFR, PDGFR, and OCTN2 shows the role of NOX4 in the regulation of EGFR and PDGFR (top); (**b**) co-expression of EGFR and PDGFR with other crucial targets responsible for initiating several types of cell proliferation diseases (bottom).

To evaluate the functional relationships among these four target receptors, we performed protein–protein interaction (PPI) network analysis using the STRING database (Figure 10).

The network analysis revealed significant functional clustering of these proteins, with NOX4 occupying a central regulatory position. NOX4 demonstrated direct and indirect interactions with EGFR and PDGFR, consistent with its role in modulating receptor tyrosine kinase signaling through reactive oxygen species (ROS) production. The network further illustrated that NOX4 influences the expression of key immunomodulatory cytokines and immune checkpoint proteins, including IL-8 and PD-L1, which are critical mediators of tumor immune evasion and therapeutic resistance. EGFR and PDGFR exhibited extensive co-expression patterns with multiple downstream effector proteins involved in cell proliferation, survival, and oncogenic transformation, including components of the PI3K/AKT, MAPK/ERK, and JAK/STAT signaling pathways (Figure 10b). OCTN2 (SLC22A5), while less densely connected compared to the receptor tyrosine kinases, showed functional associations related to metabolic regulation and drug transport.

By examining four interconnected receptors (NOX4, EGFR, PDGFR, and OCTN2) that are co-expressed and functionally linked in cancer pathogenesis, we provide systems-level insights into how single lipopeptide compounds might simultaneously modulate multiple pathways involved in oxidative stress and tumor progression. Their interconnectedness supports the hypothesis that lipopeptides capable of simultaneously binding multiple nodes within this network could lead to pleiotropic effects relevant to cancer progression and oxidative stress modulation.

## 4. Discussion

### 4.1. Plipastatin and Fengycin Significant Binding Activity

Compounds used in our modeling—bacillomycin, fengycin, fusaricidin A, fusaricidin B, iturin C, iturin D, mycosubtilin, plipastatin, polymixin B, and surfactin—are short peptides obtained from *Bacillus* that have been largely reported to exhibit antibiotic and antifungal action.

The docking of selected short-peptide compounds demonstrated promising results. For instance, iturin D was among the top-ranked compounds with NOX4 and PDGFR with binding energies of −7.85 ± 0.05 and −8.76 ± 0.09 kcal/mol, respectively. However, the free binding energy ∆G was −17.996 and −21.11 kcal/mol for NOX4 and PDGFR, respectively. Interestingly, iturin C also had −7.65 ± 0.06 kcal/mol of binding energy with NOX4 and −8.56 ± 0.05 kcal/mol with PDGFR, with a ∆G of −18.70 and −19.49 kcal/mol. Against NOX4, the three top-ranked compounds did not have better ∆G than fengycin (−28.21 kcal/mol). Further, fengycin obtained free energy of −24.51 kcal/mol with PDGFR, indicating its potential binding energy against two key receptors.

Though the binding energy of iturin C and D with NOX4 and PDGFR scored better, the binding energy seemed limited compared with fengycin. Moreover, fengycin was reported as the second top-ranked with EGFR and OCTN2, with a binding energy of −9.76 ± 0.5 and −6.75 ± 0.04 kcal/mol, and ∆G of −21.97 and −24.35 kcal/mol, respectively. Molecular docking confirmed that both the reference inhibitor, imatinib, and the investigated lipopeptide, iturin D, successfully engaged the ATP-binding pocket of PDGFR. Analysis of the binding poses revealed a significant overlap in their interaction profiles, with both ligands forming critical contacts with key residues Phe837 and Asp836 within the active site. Despite this shared binding locus, the energetics and specific interactions differed. The computed binding energy for iturin D was −8.76 ± 0.09 kcal/mol, which was less favorable than the −11.28 kcal/mol recorded for imatinib [53]. However, this was counterbalanced by iturin D’s ability to form a more extensive network of hydrogen bonds (6 versus 2–3 for imatinib) with adjacent residues. This suggested that while iturin D may have a lower overall binding energy, it achieved target engagement through a distinct and highly specific interaction pattern, potentially leading to a different mode of inhibition.

Another promising compound was plipastatin. It was top-ranked against EGFR with −11.12 ± 0.5 kcal/mol, whereas fengycin was −9.76 ± 0.5 kcal/mol at second position in terms of binding energy. It was the second top-ranked against NOX4 with a binding energy of −7.75 ± 0.03 kcal/mol and ∆G of −23.20 kcal/mol, exhibiting its potential for binding as well as binding energy. Interestingly, it was ranked second against OCTN2 with its binding energy of −8.7 ± 0.05 kcal/mol with a significant ∆G of −28.95 kcal/mol.

Overall, these compounds seem to be the most promising in this study. Plipastatin and fengycin have shown consistency in the binding energy as well as free energy, with all the receptors exhibiting significant biological activity against these receptors. They predominantly formed H-bonds, supported by the π-σ and π-alkyl interactions, which may indicate the reversible nature of these interactions.

### 4.2. Properties and Potential of Studied Bacterial Metabolites

Recently, probiotics have attracted significant attention for their potential anticancer properties. Research indicates that various strains of probiotics can play a role in cancer prevention and treatment support [54]. Probiotics can induce apoptosis (programmed cell death) in cancer cells [55]; they have been found to inhibit tumor cell proliferation and metastasis [56]. In animal models, certain strains, like *Bacillus polyfermenticus*, have demonstrated the ability to significantly suppress tumor development [57].

The anticancer effects of probiotics are often attributed to their metabolites, which can modulate cancer signaling pathways, induce autophagy, and inhibit mutagenic activity, contributing to their protective effects against cancer [58].

Several studies on the anticancer properties of *Bacillus* lipopeptides can be found, but most studies have focused on their effects through membrane disruption, mitochondrial dysfunction, ROS generation, and apoptosis induction in cancer cells [59]. Zhao et al. (2018) showed that *Bacillus subtilis* lipopeptides (a mixture of iturin forms) inhibit chronic myelogenous leukemia via paraptosis, apoptosis, and autophagy inhibition [60]. Surfactin was also active against various cancer cell types [61].

Recent computational works have explored probiotic-derived peptides targeting various receptors. Manna et al. (2020) demonstrated through *in silico* analysis that probiotic-derived lipopeptides can bind to the SARS-CoV-2 spike protein and the ACE2 receptor [62]. Patra et al. (2022) used molecular docking to show that bacterial bacteriocins interact with colorectal cancer-associated proteins COX-2, PI3K, and CASP9 [63].

Fengycins, being amphiphilic cyclic lipopeptides, consist of an anionic cyclic decapeptide with a β-hydroxy fatty acid attached at the N-terminus. Fengycins synthesized by different bacilli strains comprise some variations in their protein sequence and fatty acid chain structure [64]. Plipastatins are non-ribosomal lipodecapeptides, closely related to fengycins, and belong to the same NRP family (plipastatin family). Their assembly is encoded by operons of five synthetase genes (*ppsA-E* and *fenA-E*), which differ in whether D- or L-Tyr is incorporated at positions 3 and 9. The lipid moiety in most cases is represented by a β-hydroxy saturated fatty acid linked to the N-terminus of the peptide [65].

Fengycins and plipastatins are acknowledged as effective antifungal agents while showing low toxicity to humans and other mammals, presumably because of differences in the composition of fungal and mammalian cell membranes, the latter containing cholesterol [66]. Their anti-fungal activity comes in the form of increasing cell membrane permeability, followed by a collapse of mitochondrial membrane potential, resulting in ROS-associated cell death [67]. Apparently, if these compounds can interact with animal cells, there should be another mechanism.

Despite extensive research on lipopeptide bioactivity, we have found no studies specifically investigating the molecular interactions between *Bacillus* lipopeptides (fengycin, plipastatin, and iturins) and the transmembrane receptors NOX4, EGFR, PDGFR, and OCTN2 through computational or experimental approaches. Previous studies have not explored whether these lipopeptides can be receptor modulators beyond their well-known membrane-disrupting properties.

To our knowledge, our work is the first computational study to investigate the potential of *Bacillus* lipopeptides as direct effectors and modulators of NOX4, EGFR, PDGFR, and OCTN2. Here, we suggest an additional, previously unexplored mode of action involving specific receptor interactions. This receptor-mediated mechanism could explain some of the more subtle regulatory effects of probiotics beyond their direct cytotoxic activities.

### 4.3. Established Inhibitors and Comparative Binding Energy Analysis

To conceptualize our results, we analyzed sources on well-known inhibitors of target receptors.

Due to its role in the proliferation and malignization of tumors, NOX4 is regarded as a potential target in anticancer therapy [68]. A variety of NOX4 inhibitors are known, including extracts of natural compounds [69]. They may be a promising therapeutic strategy for various conditions, such as type 2 diabetes, diabetic retinopathy, and cardiovascular diseases. These substances can mitigate and prevent diabetes-induced beta-cell dysfunction and improve survival *in vitro* [17,70]; counteract high-fat-diet-induced glucose intolerance in animal models [70]; and improve contractile function in isolated mouse cardiomyocytes [71]. Their role has been demonstrated in the therapy of atrial fibrillation [72], idiopathic pulmonary fibrosis [73], and diabetic microangiopathy [74].

Possible inhibition of NOX4 may be a mechanism explaining the antioxidant effects demonstrated by probiotics [75,76,77] and, specifically, probiotics of the *Bacillus* genus [78,79]. It is important to note that, while NOX4 inhibitors and antioxidants aim to reduce oxidative stress, they achieve this through distinct mechanisms [17,71,80,81]. A number of authors [82,83] believe that it is essential to distinguish between oxidative eustress, which is closely related to ROS signaling, and oxidative distress, which leads to damage or even cell death. By directly inhibiting NOX4 activity, selective inhibitors can prevent pathological ROS generation linked to diseases like diabetic retinopathy, without disrupting physiological ROS signaling [69]. This targeted mechanism of action is advantageous compared to broad-spectrum antioxidant therapies that have shown limited efficacy in clinical trials.

OCTN2 is considered a target for improving the bioavailability of oral drug delivery [84], as it can influence the distribution and absorption of drugs in the lungs [85,86], anti-hepatitis B drugs in the liver [87], and the antibiotic colistin [88]. OCTN2 activity can be inhibited by anticancer drugs [29,89] and β-lactam antibiotics, which can also be its substrates [90]. OCTN2 inhibitor 3-(2,2,2-trimethylhydrazine) propionate ameliorates mitochondrial dysfunction in several conditions, such as Huntington’s disease [91]. Other OCTN2 inhibitors cause a 35% reduction in myocardial infarct size [92].

EGFR is one of the most important targets for chemotherapeutic agents aimed at treating cancerous tumors [93,94,95,96]. It is known that the selective inhibition of EGFR by gefitinib *in vivo* is directly associated with the anticancer effect of this drug [97]. EGFR is commonly targeted using monoclonal antibodies and low-molecular-weight tyrosine kinase inhibitors [98]. There are two inhibitory strategies regarding EGFR inhibition: reversible and irreversible. Inhibitors of the first type competitively bind to their ATP binding site through noncovalent interactions; second-type representatives form a covalent bond with the cysteine residue [33]. As it was demonstrated in our work, plipastatin demonstrated the possibility of binding to an active site of the molecule, but as far as these are H-bonds, we can speculate that its action can be reversible and does not belong to any of the two strategies listed above.

Studies on drugs targeting PDGFRβ have shown that reducing the expression of PDGFRβ in cells can slow down tumor progression. Imatinib is an inhibitor aimed at PDGFRβ tyrosine kinase receptors, and Marion T. Weigel and colleagues found that imatinib inhibits the growth and induces apoptosis of breast cancer cells [99]. Additionally, imatinib can enhance the antitumor effect of chemotherapy agents such as vinorelbine and paclitaxel [100]. Another research provides evidence that PDGFR inhibition in PDGFR-overexpressing cholangiocarcinoma cells leads to an increase in ROS levels and the promotion of apoptosis [101].

Thus, we can conclude that substances that interfere with these targets could provide, depending on the full biochemical and physiological context, anticancer effects or antioxidant ones, while also influencing mitochondrial dysfunction and the intracellular and intramitochondrial transport of other bioactive compounds.

A comparison between known inhibitors showed that the bacterial metabolites’ affinity is within the range of their action. The detailed comparison is presented in Table 1.

Our comparative docking analysis reveals that the *in silico* docking scores for *Bacillus* lipopeptides, obtained under our specific computational parameters, are comparable to or more favorable than the scores calculated for established pharmaceutical inhibitors under the same protocol when assessed using molecular docking approaches. It is crucial to emphasize that docking scores are protocol-dependent and serve primarily for the relative ranking of compounds within a consistent computational framework. They are not direct quantitative measures of binding affinity or biological potency, and these *in silico* predictions require experimental validation.

Molecular docking confirmed that both the reference inhibitor imatinib and the investigated lipopeptide iturin D successfully engaged the ATP-binding pocket of PDGFR. Analysis of the binding poses revealed a significant overlap in their interaction profiles, with both ligands forming critical contacts with key residues Phe837 and Asp836 within the active site. Despite this shared binding locus, the energetics and specific interactions differed. In our uniform docking protocol, the computed binding energy for iturin D was −8.76 ± 0.09 kcal/mol, while that for imatinib was −7.74 ± 0.22 kcal/mol. This suggests that iturin D exhibits a comparable, albeit slightly less favorable, *in silico* affinity for PDGFR under the same computational conditions. For reference, the literature reports for imatinib–PDGFR docking vary, with values ranging from −11.28 kcal/mol [53] to −13.1 kcal/mol [102]. However, this was counterbalanced by iturin D’s ability to form a more extensive network of hydrogen bonds (6 versus 2–3 for imatinib) with adjacent residues. This suggests that while iturin D may have a lower overall binding energy, it achieves target engagement through a distinct and highly specific interaction pattern, potentially leading to a different mode of inhibition.

A comparative analysis of binding energies was performed between the investigated *Bacillus* lipopeptides and reference inhibitors, using a consistent docking protocol to ensure direct comparability. Against EGFR, our calculated binding energy for the clinical inhibitor gefitinib was −7.78 ± 0.10 kcal/mol. In contrast, the lead lipopeptides demonstrated substantially more favorable energies: plipastatin (−11.12 ± 0.5 kcal/mol) and fengycin (−9.76 ± 0.5 kcal/mol). This indicates a more favorable *in silico* docking score for these bacterial metabolites with EGFR under our computational protocol compared to the score calculated for the established drug gefitinib under the same computational conditions. For context, the literature values for gefitinib–EGFR docking range from −7.1 to −8.0 kcal/mol [103], which aligns with our calculated result, corroborating the validity of our methodological pipeline. Similarly, the reference inhibitor erlotinib yielded a docking score of −6.48 ± 0.04 kcal/mol in our study, while the literature reports range from −9.34 to −9.72 kcal/mol [104]. In contrast, our lead compounds demonstrated substantially more favorable binding energies: plipastatin (−11.12 ± 0.5 kcal/mol) and fengycin (−9.76 ± 0.5 kcal/mol), highlighting the protocol-dependence of absolute docking scores and underscoring the importance of internal consistency for comparative analysis.

### 4.4. Hypothetical Mechanisms of Action of Bacterial Metabolites

We demonstrated that plipastatin and fengycin exhibit high binding affinities to all four receptors, with binding free energies down to −17.996 to −28.95 kcal/mol, suggesting strong and stable interactions.

Our findings propose a new molecular mechanism for the anticancer and antioxidant effects of *Bacillus* probiotics—namely, that their lipopeptide metabolites may act as direct modulators of key signaling receptors involved in oxidative stress and oncogenesis, rather than solely through non-specific membrane disruption. This receptor-mediated mechanism has not been previously proposed or investigated.

Our MD simulations data demonstrate that the predicted lipopeptide-receptor complexes remain stable over time, with protein and ligand RMSD values indicating convergent and stable binding interactions. This temporal stability assessment has not been reported in previous lipopeptide studies.

Our computational findings posit plipastatin and fengycin as pre-eminent broad-spectrum ligands, a propensity we attribute to their distinct structural architecture relative to the iturin analogs [65]. The superior binding energy and promiscuity across NOX4, EGFR, PDGFR, and OCTN2 appear to be a direct consequence of their decapeptide macrocycle, which affords a more expansive and conformationally pliable scaffold than the heptapeptide ring of the iturins [64,65]. This enhanced molecular adaptability facilitates optimal docking within disparate binding pockets, allowing for a denser network of specific interactions. Crucially, their peptide sequence is replete with polar residues, enabling an uncommonly high valency for hydrogen bonding—a feature starkly evident in our study through the formation of nine and eleven conventional H-bonds with EGFR and OCTN2, respectively. Furthermore, their amphiphilic design, featuring a β-hydroxy fatty acid tail and a substantial polar head, permits synergistic engagement with both hydrophobic niches and hydrophilic regions of the target sites, consistent with known mechanisms of lipopeptide-membrane interactions [66,105]. This results in a profoundly favorable binding free energy, as exemplified by fengycin’s remarkable ΔG of −28.21 kcal/mol with NOX4. Thus, the structural pre-adaptation of plipastatin and fengycin renders them uniquely efficacious as multi-target agents, adept at forming the stable, complex interactions requisite for modulating pivotal oncogenic and oxidative stress pathways [7,59].

Some cyclic lipopeptides produced by the *Bacillus* species can interfere with the intracellular signals of fungi and/or tumors. For instance, iturins can indirectly damage fungal cell walls by inducing mitogen-activated protein kinase Hog1 [106]. Surfactins exhibit an ability to kill breast tumor cells mediated by the disintegration of mitochondria and the activation of caspase 9 [107]. Such activity of lipopeptides is supposedly attributed to their amphipathic structure and self-assembling behavior, resulting in the formation of supramolecular complexes (such as micelles and nanofibers), which can pass through the cellular membrane and cause the degradation of the cell’s vacuome [105]. Other mechanisms of the anticancer activity of bacterial lipopeptides include increasing the permeability of the cell membrane to Ca^2+^ ions, enhancing the production of ROS, and the release of lactate dehydrogenase [7]. In addition to their cytotoxic effect, lipopeptides (such as surfactin) are capable of suppressing the expression of regulatory proteins responsible for the growth and proliferation of cancer cells, thereby reducing the tumor’s potential for invasion, migration, and metastasis [59].

It seems that, based on our findings, we can speculate that plipastatin and fengycin can also be such anticancer metabolites. Although our *in silico* results provide important theoretical insights into potential binding interactions, it needs to be emphasized that strong docking scores and high stability in MD simulation do not automatically translate to pharmacological efficacy or biological activity. Experimental validation is needed to confirm these predictions.

Thus, while the amphiphilic nature of plipastatin and fengycin undoubtedly drives their well-known membrane-perturbing effects, the deep burial within structured receptor pockets, the high-occupancy polar interaction networks, and the clear distinction from pure membrane-insertion controls observed in our simulations strongly suggest that specific receptor engagement represents a genuine and previously overlooked contributor to their biological activity. If confirmed experimentally, this dual-mode mechanism—combining non-specific membrane disruption with targeted modulation of oncogenic and redox signaling hubs—would position these *Bacillus*-derived lipopeptides as exceptionally versatile natural leads for next-generation anticancer and antioxidant therapeutics.

### 4.5. Hypotheses for Further Consideration

Thus, our molecular docking and dynamics simulations provide strong computational evidence for direct lipopeptide–receptor binding, but the functional consequences remain hypothetical. We propose three hypotheses based on our structural findings that can lay the foundation for future research in this area.

(1) Plipastatin achieves exceptional binding affinity to the EGFR ATP-binding pocket (−11.12 ± 0.5 kcal/mol), substantially exceeding gefitinib (−7.78 kcal/mol), with the interaction map showing nine conventional H-bonds directly engaging nucleotide-binding residues. Similarly, iturin D binds the PDGFR ATP-binding pocket (−8.76 ± 0.09 kcal/mol, comparable to imatinib) through critical contacts with catalytically essential residues. This binding geometry parallels established ATP-competitive tyrosine kinase inhibitors. We predict that plipastatin and iturin can inhibit EGFR and PDGFR autophosphorylation and downstream signaling (ERK1/2, AKT) in a dose-dependent manner. Testing requires purified kinase assays with IC_50_ determination, phospho-proteomic analysis in EGFR-mutant and PDGFR-dependent cell lines, and surface plasmon resonance confirmation of ATP-competitive binding with submicromolar Kd values.
(2) Iturin D shows the highest NOX4 binding affinity among all compounds tested (−7.85 ± 0.05 kcal/mol), with molecular dynamics confirming stable engagement within the ferric oxidoreductase domain. We predict this lipopeptide can directly inhibit NOX4-catalyzed H_2_O_2_ production, thereby suppressing the pathological ROS-dependent upregulation of IL-8 and PD-L1 that drives EGFR-TKI resistance in NSCLC. Combined EGFR inhibition and NOX4-mediated ROS suppression would provide synergistic anti-tumor effects in resistant cancers. This requires recombinant NOX4 assays measuring NADPH-dependent O_2_ consumption and H_2_O_2_ production, ROS quantification, and IL-8/PD-L1 expression analysis in TKI-resistant cell lines, and xenograft studies testing gefitinib plus lipopeptide versus monotherapy.
(3) Plipastatin demonstrates the highest OCTN2 binding affinity (−8.7 ± 0.05 kcal/mol), substantially exceeding carnitine itself (−4.06 kcal/mol). The interaction network involves eleven conventional H-bonds directly engaging the substrate-binding region, overlapping with residues implicated in carnitine coordination. We predict plipastatin and fengycin competitively displace carnitine and suppress carnitine-dependent fatty acid β-oxidation in OCTN2-overexpressing tumor cells. In cancers where OCTN2 promotes growth through enhanced carnitine availability, this metabolic antagonism would suppress alternative energy production in nutrient-depleted microenvironments. Testing requires recombinant OCTN2 transport assays with competitive inhibition kinetics (Lineweaver–Burk analysis), ^14^C-carnitine uptake studies in OCTN2-overexpressing cancer cell lines, metabolomic profiling of β-oxidation flux and AMPK activation upon lipopeptide treatment, and xenograft studies comparing lipopeptide monotherapy and chemotherapy combination.

Validation of these three hypotheses will require progression from *in vitro* kinase and transport assays through cell-based functional studies to *in vivo* efficacy models. Such experimental validation can determine whether the computationally predicted receptor-mediated mechanisms represent genuine contributors to the anticancer and antioxidant effects of probiotic lipopeptides.

## 5. Limitations

This study is limited to computational predictions based on molecular docking and molecular dynamics simulations. The binding affinities and interaction stabilities reported here represent theoretical predictions that will require experimental validation to make further conclusions.

A key methodological consideration is that all molecular dynamics simulations were conducted in an explicit aqueous solvent without a lipid bilayer. While this is a standard and computationally efficient approach for assessing atomic-level interactions at a defined binding site, it is a significant simplification for the studied transmembrane receptors (NOX4, EGFR, PDGFR, and OCTN2). The absence of a membrane likely influences the dynamics of the transmembrane domains and could affect the behavior of the amphiphilic lipopeptides, particularly for regions of the protein or ligand that would normally interact with a lipid interface. Consequently, our simulations primarily validate the stability of the ligand–binding site engagement in a solvated state and do not capture the full physiological context of membrane-embedded receptor regulation or potential membrane-mediated effects on binding. Future studies incorporating membrane models would provide more biologically complete insights.

Future studies should include *in vitro* binding assays (SPR, ITC), receptor activity assays (NOX4-mediated ROS production, EGFR/PDGFR tyrosine kinase activity), and cell-based functional assays (cell viability, apoptosis, ROS measurement) to confirm these *in silico* findings. Additionally, *in vivo* studies will be essential to evaluate the bioavailability, pharmacokinetics, and therapeutic efficacy of these lipopeptides.

Because amphiphilic lipopeptides can exert nonspecific membrane-disruptive effects, computational docking alone cannot confirm biological efficacy or receptor selectivity. The interactions reported here should therefore be considered predictive and require biochemical, cellular, and biophysical validation to determine their functional relevance.

## 6. Conclusions

This study provides the first *in silico* evidence suggesting that *Bacillus*-derived lipopeptides, especially plipastatin and fengycin, may interact with transmembrane receptors NOX4, EGFR, PDGFR, and OCTN2. These receptors play an important role in the development of tumors, as well as in maintaining prooxidant/antioxidant balance, mitochondrial viability, and drug bioavailability. Therefore, it can be inferred that the ability of bacterial metabolites to bind to them may be a phenomenon that enhances our understanding of the possible mechanisms of action of probiotics. These computational findings serve as a foundation for future experimental validation to determine whether these predicted interactions translate to functional biological effects relevant to oxidative stress modulation and anticancer activity. While *in vitro* and *in vivo* studies are needed to validate whether the antitumor activity of the *Bacillus* strains is related to the inhibition of the described receptors, their overall activity makes them and their metabolites, particularly NRPs, promising objects in future studies.

## Figures and Tables

**Table 1:** Binding energy of target-protein inhibitors.

Receptor	Ligand	Type of Ligand	Computational Method	Calculated in This Study Binding Energy, kcal/mol
NOX4	Fengycin	*Bacillus* lipopeptide	Docking	−7.38 ± 0.04
	Plipastatin	*Bacillus* lipopeptide	Docking	−7.75 ± 0.03
	GLX351322 [70]	A selective inhibitor of NADPH oxidase 4	Docking	−6.47 ± 0.06
EGFR	Plipastatin	*Bacillus* lipopeptide	Docking	−11.12 ± 0.50
	Fengycin	*Bacillus* lipopeptide	Docking	−9.76 ± 0.50
	Gefitinib [36]	FDA-approved TKI	Docking	−7.78 ± 0.10
	Erlotinib [36]	FDA-approved TKI	Docking	−6.48 ± 0.04
PDGFR	Fengycin	*Bacillus* lipopeptide	Docking	−8.1 ± 0.04
	Imatinib [100]	FDA-approved TKI	Docking	−7.74 ± 0.22
OCTN2	Plipastatin	*Bacillus* lipopeptide	Docking	−8.7 ± 0.05
	Fengycin	*Bacillus* lipopeptide	Docking	−6.75 ± 0.04
	Carnitine [91]	A natural substrate	Docking	−4.06 ± 0.06

## Data Availability

The key data presented in this study, including topology/parameter files for the molecular dynamics (MD) systems, and representative MD output trajectories, have been deposited in the Zenodo repository under the accession https://doi.org/10.5281/zenodo.18094800. The canonical structures of the lipopeptides are available via the PubChem Compound IDs provided in Section 2.2.

## Supplementary Materials

The following supporting information can be downloaded at: https://www.mdpi.com/article/10.3390/biotech15010004/s1, Figure S1: Q9NPH5 interactions with (a) iturin D, (b) plipastatin, and (c) iturin C can be monitored throughout the simulation. Protein-ligand interactions (or contacts) are categorized into four types: Hydrogen Bonds, Hydrophobic, Ionic and Water Bridges; Figure S2: A timeline representation of (a) iturin D–Q9NPH5, (b) plipastatin–Q9NPH5, and (c) iturin C–Q9NPH5 interactions and contacts (H-bonds, Hydrophobic, Ionic, Water bridges); Figure S3: Protein RMSF plot for 200 nsec depicting complex stability for (a) iturin D, and (b) iturin C with Q9NPH5. On this plot, peaks indicate areas of the protein that fluctuate the most during the sim-ulation. Protein residues that interact with the ligand are marked with green-colored vertical bars; Figure S4: Ligand RMSF plot for 200 nsec depicting complex stability for (a) iturin D, and (b) iturin C with Q9NPH5. Ligand RMSF shows the ligand’s fluctuations broken down by atom, corresponding to the 2D structure in the top panel; Figure S5: Iturin D docked with Q9NPH5, keeping exhaustiveness 8 and 32, pose aligned Iturin D and Q9NPH5docking with 8 exhaustiveness (left) 32 exhaustiveness (right); Figure S6: Plipastatin poses with previous docking with exhaustiveness 8 (left) aligned with redock-ing with exhaustiveness 32 (right); Figure S7: Docked poses with previous docking with exhaustiveness 8 (left) aligned with redocking with exhaustiveness 32 (right), both poses aligned in the binding cavity of the PDGFR; Figure S8: Docked poses of Bacillomycin with OCTN2; previous docking with exhaustiveness 8 (left) aligned with redocking with exhaustiveness 32 (right), both poses aligned in the binding cavity of the PDGFR; Figure S9: Re-docking of co-crystal ligands, with 1M17; Table S1: Docking score of peptide molecules against Q9NPH5; Table S2: Molecular interactions between plipastatin and the Q9NPH5 binding site; Table S3: Molecular interactions between Iturin D and the Q9NPH5 binding site; Table S4: Molecular interactions between Iturin C and the Q9NPH5 binding site; Table S5: Docking score of peptide molecules against 1M17; Table S6: Molecular interactions between plipastatin and the 1M17 binding site; Table S7: Molecular interactions between fengycin and the 1M17 binding site; Table S8: Molecular interactions between polymixin B and the 1M17 binding site; Table S9: Docking score of peptide molecules against 5GRN; Table S10: Molecular interactions between iturin D and the 5GRN binding site; Table S11: Molecular interactions between fusaricidin B and the 5GRN binding site; Table S12: Molecular interactions between fengycin and the 5GRN binding site; Table S13: Docking score of peptide molecules against O76082; Table S14: Molecular interactions between bacillomycin and the O76082 binding site; Table S15: Molecular interactions between plipastatin and the O76082 binding site; Table S16: Molecular interactions between fengycin and the O76082 binding site; Table S17: Molecular interactions between fusaricidin A and the O76082 binding site; Table S18: Detailed parameters for the 200-ns molecular dynamics simulations of the NOX4 (Q9NPH5)–lipopeptide complexes; Table S19: Configuration used; Table S20: Interaction of co-crystal ligand of 1M17 in native crystal structure; Table S21: Redocking of co-crystal Ligands with 1M17.

## Abbreviations

The following abbreviations are used in this manuscript:

NRPs	Non-ribosomal peptides
ROS	Reactive oxygen species
NOX4	NADPH oxidase 4
EGFR	Epidermal growth factor receptor
PDGFR	Platelet-derived growth factor receptor
OCTN2	Organic cation/carnitine transporter novel 2
NSCLC	Non-small-cell lung cancer
EGF	Epidermal growth factor
PDGF	Platelet-derived growth factor
TKIs	Tyrosine kinase inhibitors
PPI	Protein–protein interaction
RMSD	Root mean square deviation
MD	Molecular dynamics
∆G	Change in Gibbs free energy (binding free energy)
HCV	Hepatitis C virus
H-bonds	Hydrogen bonds

## References

[1] Zalila-Kolsi I., Ben Mahmoud A., Ali H., Sellami S., Nasfi Z., Tounsi S., Jamoussi K. (2016). Antagonist effects of *Bacillus* spp. strains against *Fusarium graminearum* for protection of durum wheat (*Triticum turgidum* L. subsp. *durum*). Microbiol. Res..

[2] Biswas I., Das Mohapatra P.K. (2023). Recent advancement in metabiotics: A consortium with bioactive molecules after fermentation by probiotic bacteria with multidisciplinary application potential and future solution in health sector. Bioresour. Technol. Rep..

[3] Ranjan A., Arora J., Chauhan A., Basniwal R.K., Kumari A., Rajput V.D., Prazdnova E.V., Ghosh A., Mukerjee N., Mandzhieva S.S. (2024). Advances in characterization of probiotics and challenges in industrial application. Biotechnol. Genet. Eng. Rev..

[4] Betzel C., Singh T.P., Visanji M., Peters K., Fittkau S., Saenger W., Wilson K.S. (1993). Structure of the complex of proteinase K with a substrate analogue hexapeptide inhibitor at 2.2-A resolution. J. Biol. Chem..

[5] Zhao J., Zhao F., Yuan J., Liu H., Wang Y. (2023). Gut microbiota metabolites, redox status, and the related regulatory effects of probiotics. Heliyon.

[6] Vo T.T.T., Lee C.W., Wu C.Z., Liu J.F., Lin W.N., Chen Y.L., Hsu L.F., Tsai M.H., Lee I.T. (2020). Surfactin from *Bacillus subtilis* attenuates ambient air particulate matter-promoted human oral cancer cells metastatic potential. J. Cancer.

[7] Yin H., Guo C., Wang Y., Liu D., Lv Y., Lv F., Lu Z. (2013). Fengycin inhibits the growth of the human lung cancer cell line 95D through reactive oxygen species production and mitochondria-dependent apoptosis. Anti-Cancer Drugs.

[8] Vo T.T.T., Wee Y., Cheng H.C., Wu C.Z., Chen Y.L., Tuan V.P., Liu J.F., Lin W.N., Lee I.T. (2021). Surfactin induces autophagy, apoptosis, and cell cycle arrest in human oral squamous cell carcinoma. Oral Dis..

[9] May J.J., Wendrich T.M., Marahiel M.A. (2001). The dhb operon of *Bacillus subtilis* encodes the biosynthetic template for the catecholic siderophore 2,3-dihydroxybenzoate-glycine-threonine trimeric ester bacillibactin. J. Biol. Chem..

[10] Dimopoulou A., Theologidis I., Benaki D., Koukounia M., Zervakou A., Tzima A., Diallinas G., Hatzinikolaou D.G., Skandalis N. (2021). Direct Antibiotic Activity of Bacillibactin Broadens the Biocontrol Range of *Bacillus amyloliquefaciens* MBI600. mSphere.

[11] Zhou M., Liu F., Yang X., Jin J., Dong X., Zeng K.W., Liu D., Zhang Y., Ma M., Yang D. (2018). Bacillibactin and Bacillomycin Analogues with Cytotoxicities against Human Cancer Cell Lines from Marine *Bacillus* sp. PKU-MA00093 and PKU-MA00092. Mar. Drugs.

[12] Carnero A. (2012). MAP17 and the double-edged sword of ROS. Biochim. Biophys. Acta.

[13] Liu W.J., Wang L., Zhou F.M., Liu S.W., Wang W., Zhao E.J., Yao Q.J., Li W., Zhao Y.Q., Shi Z. (2023). Elevated NOX4 promotes tumorigenesis and acquired EGFR-TKIs resistance via enhancing IL-8/PD-L1 signaling in NSCLC. Drug Resist. Updates.

[14] Papierniak-Wygladala A., Lamch W., Jurewicz E., Nalecz K.A. (2023). The activity and surface presence of organic cation/carnitine transporter OCTN2 (SLC22A5) in breast cancer cells depends on AKT kinase. Arch. Biochem. Biophys..

[15] Bi Y., Lei X., Chai N., Linghu E. (2021). NOX4: A potential therapeutic target for pancreatic cancer and its mechanism. J. Transl. Med..

[16] Konate M.M., Antony S., Doroshow J.H. (2020). Inhibiting the Activity of NADPH Oxidase in Cancer. Antioxid. Redox Signal..

[17] Elksnis A., Welsh N., Wikstrom P., Lau J., Carlsson P.O. (2023). The selective NOX4 inhibitor GLX7013159 decreases blood glucose concentrations and human beta-cell apoptotic rates in diabetic NMRI nu/nu mice transplanted with human islets. Free Radic. Res..

[18] Gill P.S., Wilcox C.S. (2006). NADPH Oxidases in the Kidney. Antioxid. Redox Signal..

[19] Zhang C., Hu J.J., Xia M., Boini K.M., Brimson C.A., Laperle L.A., Li P.L. (2010). Protection of podocytes from hyperhomocysteinemia-induced injury by deletion of the gp91phox gene. Free Radic. Biol. Med..

[20] Abais J.M., Zhang C., Xia M., Liu Q., Gehr T.W., Boini K.M., Li P.L. (2013). NADPH oxidase-mediated triggering of inflammasome activation in mouse podocytes and glomeruli during hyperhomocysteinemia. Antioxid. Redox Signal..

[21] Boudreau H.E., Emerson S.U., Korzeniowska A., Jendrysik M.A., Leto T.L. (2009). Hepatitis C virus (HCV) proteins induce NADPH oxidase 4 expression in a transforming growth factor beta-dependent manner: A new contributor to HCV-induced oxidative stress. J. Virol..

[22] de Mochel N.S., Seronello S., Wang S.H., Ito C., Zheng J.X., Liang T.J., Lambeth J.D., Choi J. (2010). Hepatocyte NAD(P)H oxidases as an endogenous source of reactive oxygen species during hepatitis C virus infection. Hepatology.

[23] Helfinger V., Freiherr von Gall F., Henke N., Kunze M.M., Schmid T., Rezende F., Heidler J., Wittig I., Radeke H.H., Marschall V. (2021). Genetic deletion of Nox4 enhances cancerogen-induced formation of solid tumors. Proc. Natl. Acad. Sci. USA.

[24] Mushtaq U., Bashir M., Nabi S., Khanday F.A. (2021). Epidermal growth factor receptor and integrins meet redox signaling through P66shc and Rac1. Cytokine.

[25] He Q., Kong L., Shi W., Ma D., Liu K., Yang S., Xin Q., Jiang C., Wu J. (2023). Ezetimibe inhibits triple-negative breast cancer proliferation and promotes cell cycle arrest by targeting the PDGFR/AKT pathway. Heliyon.

[26] Zheng J., Huang J., Xiao Y., Wu X., Wang K., Feng C., Gao K. (2019). Rearrangement of PDGFRβ gene in a patient with Ph-negative chronic myeloid leukemia t(5;12)(q33;p13) in imatinib mesylate treatment-free remission: A case report. Int. J. Clin. Exp. Pathol..

[27] Corless C.L., Schroeder A., Griffith D., Town A., McGreevey L., Harrell P., Shiraga S., Bainbridge T., Morich J., Heinrich M.C. (2005). PDGFRA mutations in gastrointestinal stromal tumors: Frequency, spectrum and *in vitro* sensitivity to imatinib. J. Clin. Oncol..

[28] Yang T., Liang N., Zhang J., Bai Y., Li Y., Zhao Z., Chen L., Yang M., Huang Q., Hu P. (2023). OCTN2 enhances PGC-1α-mediated fatty acid oxidation and OXPHOS to support stemness in hepatocellular carcinoma. Metab.-Clin. Exp..

[29] Juraszek B., Nalecz K.A. (2019). SLC22A5 (OCTN2) Carnitine Transporter-Indispensable for Cell Metabolism, a Jekyll and Hyde of Human Cancer. Molecules.

[30] Wang C., Uray I.P., Mazumdar A., Mayer J.A., Brown P.H. (2012). SLC22A5/OCTN2 expression in breast cancer is induced by estrogen via a novel intronic estrogen-response element (ERE). Breast Cancer Res. Treat..

[31] Sun D., Chen Q., Gai Z., Zhang F., Yang X., Hu W., Chen C., Yang G., Hörmann S., Kullak-Ublick G.A. (2021). The Role of the Carnitine/Organic Cation Transporter Novel 2 in the Clinical Outcome of Patients With Locally Advanced Esophageal Carcinoma Treated with Oxaliplatin. Front. Pharmacol..

[32] Thannickal V.J., Jandeleit-Dahm K., Szyndralewiez C., Török N.J. (2023). Pre-clinical evidence of a dual NADPH oxidase 1/4 inhibitor (setanaxib) in liver, kidney and lung fibrosis. J. Cell. Mol. Med..

[33] Abourehab M.A.S., Alqahtani A.M., Youssif B.G.M., Gouda A.M. (2021). Globally Approved EGFR Inhibitors: Insights into Their Syntheses, Target Kinases, Biological Activities, Receptor Interactions, and Metabolism. Molecules.

[34] Kou L., Yao Q., Sivaprakasam S., Luo Q., Sun Y., Fu Q., He Z., Sun J., Ganapathy V. (2017). Dual targeting of l-carnitine-conjugated nanoparticles to OCTN2 and ATB(0,+) to deliver chemotherapeutic agents for colon cancer therapy. Drug Deliv..

[35] Kitchen D.B., Decornez H., Furr J.R., Bajorath J. (2004). Docking and scoring in virtual screening for drug discovery: Methods and applications. Nat. Rev. Drug Discov..

[36] Lionta E., Spyrou G., K Vassilatis D., Cournia Z. (2014). Structure-based virtual screening for drug discovery: Principles, applications and recent advances. Curr. Top. Med. Chem..

[37] Ferreira L.G., Dos Santos R.N., Oliva G., Andricopulo A.D. (2015). Molecular docking and structure-based drug design strategies. Molecules.

[38] Meng X.-Y., Zhang H.-X., Mezei M., Cui M. (2011). Molecular docking: A powerful approach for structure-based drug discovery. Curr. Comput.-Aided Drug Des..

[39] Evans D.A. (2014). History of the Harvard ChemDraw project. Angew. Chem..

[40] Trott O., Olson A.J. (2010). AutoDock Vina: Improving the speed and accuracy of docking with a new scoring function, efficient optimization, and multithreading. J. Comput. Chem..

[41] Takac I., Schröder K., Zhang L., Lardy B., Anilkumar N., Lambeth J.D., Shah A.M., Morel F., Brandes R.P. (2011). The E-loop is involved in hydrogen peroxide formation by the NADPH oxidase Nox4. J. Biol. Chem..

[42] Park H.S., Jung H.Y., Park E.Y., Kim J., Lee W.J., Bae Y.S. (2004). Cutting edge: Direct interaction of TLR4 with NAD(P)H oxidase 4 isozyme is essential for lipopolysaccharide-induced production of reactive oxygen species and activation of NF-kappa B. J. Immunol..

[43] Jura N., Endres N.F., Engel K., Deindl S., Das R., Lamers M.H., Wemmer D.E., Zhang X., Kuriyan J. (2009). Mechanism for activation of the EGF receptor catalytic domain by the juxtamembrane segment. Cell.

[44] Yun C.H., Boggon T.J., Li Y., Woo M.S., Greulich H., Meyerson M., Eck M.J. (2007). Structures of lung cancer-derived EGFR mutants and inhibitor complexes: Mechanism of activation and insights into differential inhibitor sensitivity. Cancer Cell.

[45] Vassbotn F.S., Havnen O.K., Heldin C.H., Holmsen H. (1994). Negative feedback regulation of human platelets via autocrine activation of the platelet-derived growth factor alpha-receptor. J. Biol. Chem..

[46] Yu J.C., Heidaran M.A., Pierce J.H., Gutkind J.S., Lombardi D., Ruggiero M., Aaronson S.A. (1991). Tyrosine mutations within the alpha platelet-derived growth factor receptor kinase insert domain abrogate receptor-associated phosphatidylinositol-3 kinase activity without affecting mitogenic or chemotactic signal transduction. Mol. Cell. Biol..

[47] Zhang L., Gui T., Console L., Scalise M., Indiveri C., Hausler S., Kullak-Ublick G.A., Gai Z., Visentin M. (2021). Cholesterol stimulates the cellular uptake of L-carnitine by the carnitine/organic cation transporter novel 2 (OCTN2). J. Biol. Chem..

[48] Wagner C.A., Lükewille U., Kaltenbach S., Moschen I., Bröer A., Risler T., Bröer S., Lang F. (2000). Functional and pharmacological characterization of human Na(+)-carnitine cotransporter hOCTN2. Am. J. Physiol.-Ren. Physiol..

[49] Morris G.M., Huey R., Lindstrom W., Sanner M.F., Belew R.K., Goodsell D.S., Olson A.J. (2009). AutoDock4 and AutoDockTools4: Automated docking with selective receptor flexibility. J. Comput. Chem..

[50] BIOVIA Discovery Studio Visualizer (2017). Discovery Studio Visualizer.

[51] Bowers K.J., Chow E., Xu H., Dror R.O., Eastwood M.P., Gregersen B.A., Klepeis J.L., Kolossvary I., Moraes M.A., Sacerdoti F.D. Scalable algorithms for molecular dynamics simulations on commodity clusters. Proceedings of the 2006 ACM/IEEE Conference on Supercomputing.

[52] von Löhneysen K., Noack D., Wood M.R., Friedman J.S., Knaus U.G. (2010). Structural insights into Nox4 and Nox2: Motifs involved in function and cellular localization. Mol. Cell. Biol..

[53] Padhy G.K., Behera S.K., Biswas A., Raul S.K., Panda J. (2025). Research Article *In silico* Investigation of Ligand Binding Mechanisms in PDGFR-A. Res. J. Pharm. Life Sci. Vol..

[54] Slizewska K., Markowiak-Kopec P., Slizewska W. (2020). The Role of Probiotics in Cancer Prevention. Cancers.

[55] Gholipour F., Amini M., Baradaran B., Mokhtarzadeh A., Eskandani M. (2023). Anticancer properties of curcumin-treated *Lactobacillus plantarum* against the HT-29 colorectal adenocarcinoma cells. Sci. Rep..

[56] Khaledi M., Esmaeili Gouvarchin Ghaleh H., Abyazi M.A., Mahmoodzadeh Hosseini H., Golmohammadi R. (2023). The Anticancer Properties of Probiotic Species. J. Appl. Biotechnol. Rep..

[57] Lu K., Dong S., Wu X., Jin R., Chen H. (2021). Probiotics in Cancer. Front. Oncol..

[58] Sankarapandian V., Venmathi Maran B.A., Rajendran R.L., Jogalekar M.P., Gurunagarajan S., Krishnamoorthy R., Gangadaran P., Ahn B.C. (2022). An Update on the Effectiveness of Probiotics in the Prevention and Treatment of Cancer. Life.

[59] Dan A.K., Manna A., Ghosh S., Sikdar S., Sahu R., Parhi P.K., Parida S. (2021). Molecular mechanisms of the lipopeptides from *Bacillus subtilis* in the apoptosis of cancer cells-A review on its current status in different cancer cell lines. Adv. Cancer Biol.-Metast..

[60] Zhao H., Yan L., Xu X., Jiang C., Shi J., Zhang Y., Liu L., Lei S., Shao D., Huang Q. (2018). Potential of *Bacillus subtilis* lipopeptides in anti-cancer I: Induction of apoptosis and paraptosis and inhibition of autophagy in K562 cells. AMB Express.

[61] Wu Y.-S., Ngai S.-C., Goh B.-H., Chan K.-G., Lee L.-H., Chuah L.-H. (2017). Anticancer Activities of Surfactin and Potential Application of Nanotechnology Assisted Surfactin Delivery. Front. Pharmacol..

[62] Manna S., Chowdhury T., Chakraborty R., Mandal S.M. (2021). Probiotics-Derived Peptides and Their Immunomodulatory Molecules Can Play a Preventive Role Against Viral Diseases Including COVID-19. Probiot. Antimicrob. Proteins.

[63] Patra S., Sahu N., Saxena S., Pradhan B., Nayak S.K., Roychowdhury A. (2022). Effects of Probiotics at the Interface of Metabolism and Immunity to Prevent Colorectal Cancer-Associated Gut Inflammation: A Systematic Network and Meta-Analysis With Molecular Docking Studies. Front. Microbiol..

[64] Sur S., Romo T.D., Grossfield A. (2018). Selectivity and Mechanism of Fengycin, an Antimicrobial Lipopeptide, from Molecular Dynamics. J. Phys. Chem. B.

[65] Hussein W. (2019). Fengycin or plipastatin? A confusing question in Bacilli. Bio Technol..

[66] Sur S., Grossfield A. (2022). Effects of cholesterol on the mechanism of fengycin, a biofungicide. Biophys. J..

[67] Zhang L., Sun C. (2018). Fengycins, Cyclic Lipopeptides from Marine *Bacillus subtilis* Strains, Kill the Plant-Pathogenic Fungus *Magnaporthe grisea* by Inducing Reactive Oxygen Species Production and Chromatin Condensation. Appl. Environ. Microbiol..

[68] Gong S., Wang S., Shao M. (2022). NADPH Oxidase 4: A Potential Therapeutic Target of Malignancy. Front. Cell Dev. Biol..

[69] Dionysopoulou S., Wikstrom P., Bucolo C., Romano G.L., Micale V., Svensson R., Spyridakos D., Mastrodimou N., Georgakis S., Verginis P. (2023). Topically Administered NOX4 Inhibitor, GLX7013114, Is Efficacious in Treating the Early Pathological Events of Diabetic Retinopathy. Diabetes.

[70] Anvari E., Wikstrom P., Walum E., Welsh N. (2015). The novel NADPH oxidase 4 inhibitor GLX351322 counteracts glucose intolerance in high-fat diet-treated C57BL/6 mice. Free Radic. Res..

[71] Szekeres F.L.M., Walum E., Wikstrom P., Arner A. (2021). A small molecule inhibitor of Nox2 and Nox4 improves contractile function after ischemia-reperfusion in the mouse heart. Sci. Rep..

[72] Ramos-Mondragon R., Lozhkin A., Vendrov A.E., Runge M.S., Isom L.L., Madamanchi N.R. (2023). NADPH Oxidases and Oxidative Stress in the Pathogenesis of Atrial Fibrillation. Antioxidants.

[73] Sylvester A.L., Zhang D.X., Ran S., Zinkevich N.S. (2022). Inhibiting NADPH Oxidases to Target Vascular and Other Pathologies: An Update on Recent Experimental and Clinical Studies. Biomolecules.

[74] Wang D., Li J., Luo G., Zhou J., Wang N., Wang S., Zhao R., Cao X., Ma Y., Liu G. (2023). Nox4 as a novel therapeutic target for diabetic vascular complications. Redox Biol..

[75] Mishra V., Shah C., Mokashe N., Chavan R., Yadav H., Prajapati J. (2015). Probiotics as potential antioxidants: A systematic review. J. Agric. Food Chem..

[76] Musazadeh V., Faghfouri A.H., Zarezadeh M., Pakmehr A., Moghaddam P.T., Hamedi-Kalajahi F., Jahandideh A., Ghoreishi Z. (2023). Remarkable impacts of probiotics supplementation in enhancing of the antioxidant status: Results of an umbrella meta-analysis. Front. Nutr..

[77] Wang Y., Wu Y., Wang Y., Xu H., Mei X., Yu D., Wang Y., Li W. (2017). Antioxidant Properties of Probiotic Bacteria. Nutrients.

[78] Kodali V.P., Sen R. (2008). Antioxidant and free radical scavenging activities of an exopolysaccharide from a probiotic bacterium. Biotechnol. J..

[79] Safronova L.S., Skorochod I.A., Ilyash V.M. (2021). Antioxidant and Antiradical Properties of Probiotic Strains *Bacillus amyloliquefaciens* ssp. plantarum. Probiot. Antimicrob. Proteins.

[80] Altenhofer S., Radermacher K.A., Kleikers P.W., Wingler K., Schmidt H.H. (2015). Evolution of NADPH Oxidase Inhibitors: Selectivity and Mechanisms for Target Engagement. Antioxid. Redox Signal..

[81] Dao V.T., Elbatreek M.H., Altenhofer S., Casas A.I., Pachado M.P., Neullens C.T., Knaus U.G., Schmidt H. (2020). Isoform-selective NADPH oxidase inhibitor panel for pharmacological target validation. Free Radic. Biol. Med..

[82] Okegbe C., Sakhtah H., Sekedat M.D., Price-Whelan A., Dietrich L.E. (2012). Redox eustress: Roles for redox-active metabolites in bacterial signaling and behavior. Antioxid. Redox Signal..

[83] Selye H. (1975). Stress and distress. Compr. Ther..

[84] Wang G., Zhao L., Jiang Q., Sun Y., Zhao D., Sun M., He Z., Sun J., Wang Y. (2020). Intestinal OCTN2- and MCT1-targeted drug delivery to improve oral bioavailability. Asian J. Pharm. Sci..

[85] Mukherjee M., Latif M.L., Pritchard D.I., Bosquillon C. (2013). In-cell Western detection of organic cation transporters in bronchial epithelial cell layers cultured at an air-liquid interface on Transwell((R)) inserts. J. Pharmacol. Toxicol. Methods.

[86] Nakamura T., Nakanishi T., Haruta T., Shirasaka Y., Keogh J.P., Tamai I. (2010). Transport of ipratropium, an anti-chronic obstructive pulmonary disease drug, is mediated by organic cation/carnitine transporters in human bronchial epithelial cells: Implications for carrier-mediated pulmonary absorption. Mol. Pharm..

[87] Yang X., Ma Z., Zhou S., Weng Y., Lei H., Zeng S., Li L., Jiang H. (2016). Multiple Drug Transporters Are Involved in Renal Secretion of Entecavir. Antimicrob. Agents Chemother..

[88] Visentin M., Gai Z., Torozi A., Hiller C., Kullak-Ublick G.A. (2017). Colistin is substrate of the carnitine/organic cation transporter 2 (OCTN2, SLC22A5). Drug Metab. Dispos..

[89] Hu C., Lancaster C.S., Zuo Z., Hu S., Chen Z., Rubnitz J.E., Baker S.D., Sparreboom A. (2012). Inhibition of OCTN2-mediated transport of carnitine by etoposide. Mol. Cancer Ther..

[90] Ganapathy M.E., Huang W., Rajan D.P., Carter A.L., Sugawara M., Iseki K., Leibach F.H., Ganapathy V. (2000). β-lactam antibiotics as substrates for OCTN2, an organic cation/carnitine transporter. J. Biol. Chem..

[91] Di Cristo F., Calarco A., Digilio F.A., Sinicropi M.S., Rosano C., Galderisi U., Melone M.A.B., Saturnino C., Peluso G. (2020). The Discovery of Highly Potent THP Derivatives as OCTN2 Inhibitors: From Structure-Based Virtual Screening to *In vivo* Biological Activity. Int. J. Mol. Sci..

[92] Liepinsh E., Makrecka M., Kuka J., Cirule H., Makarova E., Sevostjanovs E., Grinberga S., Vilskersts R., Lola D., Loza E. (2014). Selective inhibition of OCTN2 is more effective than inhibition of gamma-butyrobetaine dioxygenase to decrease the availability of l-carnitine and to reduce myocardial infarct size. Pharmacol. Res..

[93] Hirsch F.R., Scagliotti G.V., Mulshine J.L., Kwon R., Curran W.J., Wu Y.L., Paz-Ares L. (2017). Lung cancer: Current therapies and new targeted treatments. Lancet.

[94] Mok T.S., Wu Y.L., Ahn M.J., Garassino M.C., Kim H.R., Ramalingam S.S., Shepherd F.A., He Y., Akamatsu H., Theelen W.S. (2017). Osimertinib or Platinum-Pemetrexed in EGFR T790M-Positive Lung Cancer. N. Engl. J. Med..

[95] Rosell R., Carcereny E., Gervais R., Vergnenegre A., Massuti B., Felip E., Palmero R., Garcia-Gomez R., Pallares C., Sanchez J.M. (2012). Erlotinib versus standard chemotherapy as first-line treatment for European patients with advanced EGFR mutation-positive non-small-cell lung cancer (EURTAC): A multicentre, open-label, randomised phase 3 trial. Lancet Oncol..

[96] Zhou C., Wu Y.L., Chen G., Feng J., Liu X.Q., Wang C., Zhang S., Wang J., Zhou S., Ren S. (2011). Erlotinib versus chemotherapy as first-line treatment for patients with advanced EGFR mutation-positive non-small-cell lung cancer (OPTIMAL, CTONG-0802): A multicentre, open-label, randomised, phase 3 study. Lancet Oncol..

[97] He P., Jing J., Du L., Zhang X., Ren Y., Yang H., Yu B., Liu H. (2023). Discovery of YS-363 as a highly potent, selective, and orally e ffi cacious EGFR inhibitor. Biomed. Pharmacother..

[98] Sabbah D.A., Hajjo R., Sweidan K. (2020). Review on Epidermal Growth Factor Receptor (EGFR) Structure, Signaling Pathways, Interactions, and Recent Updates of EGFR Inhibitors. Curr. Top. Med. Chem..

[99] Weigel M.T., Meinhold-Heerlein I., Bauerschlag D.O., Schem C., Bauer M., Jonat W., Maass N., Mundhenke C. (2009). Combination of imatinib and vinorelbine enhances cell growth inhibition in breast cancer cells via PDGFR beta signalling. Cancer Lett..

[100] Apte S.M., Fan D., Killion J.J., Fidler I.J. (2004). Targeting the Platelet-Derived Growth Factor Receptor in Antivascular Therapy for Human Ovarian Carcinoma. Clin. Cancer Res..

[101] Duangdara J., Boonsri B., Sayinta A., Supradit K., Thintharua P., Kumkate S., Suriyonplengsaeng C., Larbcharoensub N., Mingphruedhi S., Rungsakulkij N. (2023). CP-673451, a Selective Platelet-Derived Growth Factor Receptor Tyrosine Kinase Inhibitor, Induces Apoptosis in *Opisthorchis viverrini*-Associated Cholangiocarcinoma via Nrf2 Suppression and Enhanced ROS. Pharmaceuticals.

[102] Khattabi K.E., Lemriss S., Jaoudi R.E., Zouaidia F. (2024). Molecular docking and simulation analysis of c-KIT and PDGFRα with phytochemicals as dual inhibitors for GIST. Bioinformation.

[103] Bommu U.D., Konidala K.K., Pamanji R., Yeguvapalli S. (2018). Computational screening, ensemble docking and pharmacophore analysis of potential gefitinib analogues against epidermal growth factor receptor. J. Recept. Signal Transduct..

[104] Park J.H., Liu Y., Lemmon M.A., Radhakrishnan R. (2012). Erlotinib binds both inactive and active conformations of the EGFR tyrosine kinase domain. Biochem. J..

[105] Lu J.Y., Huang W.T., Zhou K., Zhao X., Yang S., Xia L., Ding X. (2022). Microbial Lipopeptide Supramolecular Self-Assemblies as a Methuosis-Like Cell Death Inducer with *In vivo* Antitumor Activity. Small.

[106] Santos-Lima D., de Castro Spadari C., de Morais Barroso V., Carvalho J.C.S., de Almeida L.C., Alcalde F.S.C., Ferreira M.J.P., Sannomiya M., Ishida K. (2023). Lipopeptides from an isolate of *Bacillus subtilis* complex have inhibitory and antibiofilm effects on *Fusarium solani*. Appl. Microbiol. Biotechnol..

[107] Liang C., Xi-xi X., Yun-xiang S., Qiu-hua X., Yang-yong L., Yuan-sen H., Ke B. (2023). Surfactin inhibits *Fusarium graminearum* by accumulating intracellular ROS and inducing apoptosis mechanisms. World J. Microbiol. Biotechnol..

