# Supplementary material for: Lipopeptides from Bacillus Probiotics Can Target Transmembrane Receptors NOX4, EGFR, PDGFR, and OCTN2 Involved in Oxidative Stress and Oncogenesis
Source: BioTech (Basel). 2026 Jan 6;15(1):4. doi: 10.3390/biotech15010004 (PMC12821567; doi:10.3390/biotech15010004)
Supplement: Supplementary file 1 [file biotech-15-00004-s001.zip › biotech-3948141-supplementary.pdf]

BioTech

Supplementary Materials

Lipopeptides from *Bacillus* Probiotics Can Target Transmembrane Receptors NOX4, EGFR, PDGFR, and OCTN2 Involved in Oxidative Stress and Oncogenesis

Evgeniya Prazdnova, Fadi Amirdzhanov *, Anuj Ranjan and Radomir Skripnichenko

Academy of Biology and Medicine, Southern Federal University, Rostov-on-Don 344090, Russia; prazdnova@sfedu.ru (E.P.); randzhan@sfedu.ru (A.R.); radomirskr@inbox.ru (R.S.)
* Correspondence: amirdzhanov@sfedu.ru; Tel.: +7-(908)518-42-56

1. Molecular docking

1.1. Docking with NADPH Oxidase 4 (Q9NPH5)

Table S1. Docking score of peptide molecules against Q9NPH5

Ligand	Binding energy (kcal/mol)	RMSD LB	RMSD UB	Δ G (kcal/mol)
Iturin D	-7.85 ± 0.05	1.193 ± 0.05	1.153 ± 0.04	-17.996
Plipastin	-7.75 ± 0.03	1.195 ± 0.04	2.308 ± 0.05	-23.20
Iturin C	-7.65 ± 0.06	2.696 ± 0.05	2.599 ± 0.05	-18.70
Mycosubtilin	-7.62± 0.04	1.1955 ± 0.04	2.308 ± 0.06	-20.07
Fengycin	-7.38 ± 0.04	1.642 ± 0.05	2.396 ± 0.04	-28.21
Fusaricidin B	-7.04 ± 0.04	18.612 ± 0.39	2.607 ± 0.23	-17.94
Bacillomycin	-7.03 ± 0.03	3.452 ± 0.05	2.785 ± 0.04	-17.92
Surfactin	-6.71 ± 0.06	3.012 ± 0.04	2.625 ± 0.05	-19.39
Fusaricidin A	-6.63 ± 0.03	1.558 ± 0.05	2.59 ± 0.06	-16.50
Polymixin B	-5.54 ± 0.06	2.635 ± 0.04	2.745 ± 0.04	-21.72

Table S2. Molecular interactions between plipastatin and the Q9NPH5 binding site

Type of Bond	Interaction between	Distance (in Å)
Conventional Hydrogen Bond	A:TYR294:OH - A:plipastatin:O20	3.07564
Conventional Hydrogen Bond	A:plipastatin:H31 - A:ASN195:OD1	1.83718
Conventional Hydrogen Bond	A:plipastatin:H110 - A:ASN195:OD1	2.51787
Conventional Hydrogen Bond	A:plipastatin:H47 - A:SER305:OG	2.1379
Carbon Hydrogen Bond	A:ARG298:CD - A:plipastatin:O4	3.16334

Pi-Sigma	A:plipastatin:C10 - A:PHE197	3.83055
Pi-Sigma	A:plipastatin:C1 - A:PHE200	3.57715
Pi-Sulfur	A:CYS295:SG - A:plipastatin	5.67027
Pi-Alkyl	A:HIS105 - A:plipastatin	5.32117
Pi-Alkyl	A:HIS105 - A:plipastatin	5.29908
Pi-Alkyl	A:PHE197 - A:plipastatin	4.96729
Pi-Alkyl	A:PHE197 - A:plipastatin	5.36262
Pi-Alkyl	A:PHE200 - A:plipastatin	5.01217
Pi-Alkyl	A:plipastatin - A:LEU299	4.53768

Table S3. Molecular interactions between Iturin D and the Q9NPH5 binding site

**Type of Bond**	**Interaction between**	**Distance (in Å)**
Conventional Hydrogen Bond	A:ARG304:NH1 - A:Iturin D:O2	3.17763
Conventional Hydrogen Bond	A:ARG334:NH2 - A:Iturin D:O13	3.15327
Conventional Hydrogen Bond	A:Iturin D:H58 - A:GLU329:O	2.75389
Carbon Hydrogen Bond	A:TYR82:CA - A:Iturin D:O14	3.59154
Carbon Hydrogen Bond	A:GLY85:CA - A:Iturin D:O13	3.17371
Pi-Alkyl	A:TYR300 - A:Iturin D:C1	4.4779
Pi-Alkyl	A:Iturin D - A:ALA81	5.43897
Pi-Alkyl	A:TYR82 - A:Iturin D	4.31623

Table S4. Molecular interactions between Iturin C and the Q9NPH5 binding site

**Type of Bond**	**Interaction between**	**Distance (in Å)**
Conventional Hydrogen Bond	A:ARG304:NH1 - A:Iturin C:O2	3.37215
Conventional Hydrogen Bond	A:Iturin C:H62 - A:ASN330:O	2.58839
Conventional Hydrogen Bond	A:Iturin C:H58 - A:GLU329:O	2.30781
Carbon Hydrogen Bond	A:TYR82:CA - A:Iturin C:O14	3.72594
Carbon Hydrogen Bond	A:ARG304:CD - A:Iturin C:O4	3.51684
Pi-Sigma	A:Iturin C:C2 - A:TYR300	3.72662
Pi-Alkyl	A:TYR82 - A:Iturin C	3.37215

1.2. Docking with Epidermal Growth Factor Receptor Tyrosine (1M17)

Table S5. Docking score of peptide molecules against 1M17

Ligand	Binding energy (kcal/mol)	RMSD LB	RMSD UB	Δ G (kcal/mol)
Plipastatin	-11.12 ± 0.5	1.987 ± 0.04	1.678 ± 0.05	-21.34
Fengycin	-9.76 ± 0.5	2.345 ± 0.05	2.596 ± 0.05	-21.97
Ploymixin B	-7.92 ± 0.06	1.825 ± 0.04	1.596 ± 0.04	-19.87
Surfactin	-7.58 ± 0.04	2.698 ± 0.05	2.989 ± 0.06	-20.82
Bacillomycin	-7.28 ± 0.04	3.478 ± 0.78	4.875 ± 0.09	-18.01
Iturin D	-5.74 ± 0.05	4.756 ± 0.09	6.596 ± 0.14	-17.26
Iturin C	-5.71 ± 0.06	3.478 ± 0.07	4.787 ± 0.07	-18.04
Mycosubtilin	-4.47 ± 0.09	6.746 ± 0.08	4.698 ± 0.13	-21.64
Fusaricidin B	-4.85 ± 0.9	2.475± 0.07	1.745± 0.05	-17.14

Table S6. Molecular interactions between plipastatin and the 1M17 binding site

Type of Bond	Interaction between	Distance (in Å)
Conventional Hydrogen Bond	A:LYS828:NZ - A:Plipastatin:O6	3.26994
Conventional Hydrogen Bond	A:THR830:OG1 - A:Plipastatin:O16	2.65814
Conventional Hydrogen Bond	A:ASP831:N:B - A:Plipastatin:O16	2.88585
Conventional Hydrogen Bond	A:Plipastatin:H46 - A:ARG817:O	2.45985
Conventional Hydrogen Bond	A:Plipastatin:H59 - A:CYS751:N:B	2.98065
Conventional Hydrogen Bond	A:Plipastatin:H61 - A:MET742:O	2.86863
Conventional Hydrogen Bond	A:Plipastatin:H61 - A:CYS751:O	3.02195
Conventional Hydrogen Bond	A:Plipastatin:H61 - A:CYS751:O:B	3.06691
Conventional Hydrogen Bond	A:Plipastatin:H72 - A:THR830:OG1	2.52759
Carbon Hydrogen Bond	A:CYS751:C:B - A:Plipastatin:O7	3.0707
Carbon Hydrogen Bond	A:Plipastatin:C51 - A:THR830:OG1	3.70875

https://doi.org/10.3390/biotech15010004

Pi-Cation	A:Plipastatin:N7 - A:PHE832	4.0012
Pi-Anion	A:ASP746:OD1 - A:Plipastatin	2.9854
Sulfur-X	A:MET742:SD - A:Plipastatin:O13	3.26192

Table S7. Molecular interactions between fengycin and the 1M17 binding site

Type of Bond	Interaction between	Distance (in Å)
Conventional Hydrogen Bond	A:THR766:HG1 - A:fengycin_:O11	2.51428
Conventional Hydrogen Bond	A:THR830:HG1 - A:fengycin_:O10	2.12546
Conventional Hydrogen Bond	A:fengycin_:O1 - A:LYS822:O	2.30261
Conventional Hydrogen Bond	A:fengycin_:H110 - A:GLY772:O	2.91285
Conventional Hydrogen Bond	A:fengycin_:H48 - A:LYS822:O	1.4748

Table S8. Molecular interactions between polymixin B and the 1M17 binding site

Type of Bond	Interaction between	Distance (in Å)
Conventional Hydrogen Bond	A:CYS751:SG - A:Polymixin B:O6	2.6098
Conventional Hydrogen Bond	A:GLN767:N - A:Polymixin B:O7	2.4878
Conventional Hydrogen Bond	A:VAL821:N - A:Polymixin B:O12	2.77951
Conventional Hydrogen Bond	A:THR830:OG1 - A:Polymixin B:O8	2.89735
Conventional Hydrogen Bond	A:Polymixin B:H86 - A:ALA719:O	2.74637
Conventional Hydrogen Bond	A:Polymixin B:H86 - A:THR766:OG1	2.22266
Conventional Hydrogen Bond	A:Polymixin B:H87 - A:ALA719:O	2.56002
Conventional Hydrogen Bond	A:Polymixin B:H87 - A:LEU764:O	2.12905
Conventional Hydrogen Bond	A:Polymixin B:H93 - A:ILE829:O	1.92081
Carbon Hydrogen Bond	A:GLY772:CA - A:Polymixin B:O10	3.57972
Carbon Hydrogen Bond	A:Polymixin B:C20 - A:PRO717:O	3.34683

https://doi.org/10.3390/biotech15010004

Pi-Alkyl	A:PHE771 - A:Polymixin B	4.40089

1.3. Docking with Platelet-Derived Growth Factor Receptor (5GRN)

Table S9. Docking score of peptide molecules against 5GRN

**Ligand**	**Binding energy (kcal/mol)**	**RMSD LB**	**RMSD UB**	**Δ G (kcal/mol)**
Iturin D	-8.76 ± 0.09	2.206 ±0.03	3.373 ±0.07	-21.11
Iturin C	- 8.56 ± 0.05	2.224 ±0.05	3.474 ±0.07	-19.49
Fusaricidin B	- 8.34 ± 0.05	1.133 ±0.03	1.272 ±0.04	-20.63
Fusaricidin A	- 8.2 ± 0.03	20.805 ±1.92	25.470 ±0.94	-20.36
Fengycin	- 8.1 ± 0.04	2.568 ±0.06	5.891 ±0.04	-24.51
Bacillomycin	- 7.4 ± 0.07	1.388 ±0.05	1.997 ±0.05	-19.28
Polymyxin B	- 7.3 ± 0.05	2.106 ±0.07	3.591 ±0.04	-18.12
Surfactin	- 6.8 ± 1.09	4.701 ±0.93	11.983 ±1.07	-17.97

Table S10. Molecular interactions between iturin D and the 5GRN binding site

**Type of Bond**	**Interaction between**	**Distance (in Å)**
Conventional Hydrogen Bond	A:ASN684:ND2 - A:Iturin D:O1	2.85068
Conventional Hydrogen Bond	A:HIS687:ND1 - A:Iturin D:O5	3.35785
Conventional Hydrogen Bond	A:ARG841:NH1 - A:Iturin D:O14	3.35589
Conventional Hydrogen Bond	A:Iturin D:H33 - A:ASN684:OD1	2.77127
Conventional Hydrogen Bond	A:Iturin D:H73 - A:LEU599:O	2.32499
Conventional Hydrogen Bond	A:Iturin D:H70 - A:SER601:O	2.10713
Pi-Sigma	A:Iturin D:C1 - A:PHE837	3.63085
Pi-Alkyl	A:PHE837 - A:Iturin D	4.40583

Table S11. Molecular interactions between fusaricidin B and the 5GRN binding site

Type of Bond	Interaction between	Distance (in Å)
Conventional Hydrogen Bond	A:Fusaricidin B:N8 - A:ASP836:OD2	4.74187
Conventional Hydrogen Bond	A:SER643:OG - A:Fusaricidin B:O10	3.15067
Conventional Hydrogen Bond	A:Fusaricidin B:HN - A:VAL815:O	2.46719
Conventional Hydrogen Bond	A:Fusaricidin B:HN - A:HIS816:O	2.17554
Conventional Hydrogen Bond	A:Fusaricidin B:H75 - A:VAL815:O	2.58784
Conventional Hydrogen Bond	A:Fusaricidin B:H75 - A:HIS816:O	2.50716
Conventional Hydrogen Bond	A:Fusaricidin B:H24 - A:ASP836:OD1	2.77413
Conventional Hydrogen Bond	A:Fusaricidin B:H23 - A:ASP818:OD2	2.1241
Carbon Hydrogen Bond	A:Fusaricidin B:H23 - A:ASP836:OD1	2.70412
Pi-Alkyl	A:HIS816 - A:Fusaricidin B	5.42686

Table S12. Molecular interactions between fengycin and the 5GRN binding site

Type of Bond	Interaction between	Distance (in Å)
Conventional Hydrogen Bond	A:ARG597:NH2 - A:Fengycin:O1	3.39852
Conventional Hydrogen Bond	A:ASN848:ND2 - A:Fengycin:O7	3.29279
Conventional Hydrogen Bond	A:Fengycin:H37 - A:ASP681:O	3.00257
Conventional Hydrogen Bond	A:Fengycin:H37 - A:ASP681:OD1	2.30048
Conventional Hydrogen Bond	A:Fengycin:H47 - A:VAL598:O	2.45472
Conventional Hydrogen Bond	A:Fengycin:H46 - A:VAL598:O	2.7455
Carbon Hydrogen Bond	A:Fengycin:C38 - A:SER847:OG	3.38973
Pi-Donor Hydrogen Bond	A:SER847:OG - A:Fengycin	3.60006
Alkyl	A:LYS627 - A:Fengycin	5.07363
Alkyl	A:LYS627 - A:Fengycin	4.62783

https://doi.org/10.3390/biotech15010004

Pi-Alkyl	A:Fengycin - A:VAL683	5.30989
Pi-Alkyl	A:Fengycin - A:ARG822	5.45354

1.4. Docking with organic cation / carnitine transporter 2 (O76082)

Table S13. Docking score of peptide molecules against O76082

Ligand	Binding energy (kcal/mol)	RMSD LB	RMSD UB	Δ G (kcal/mol)
Bacillomycin	-9.66 ± 0.05	2.329 ± 0.06	2.655 ± 0.05	- 21.36
Plipastatin	-8.7 ± 0.05	2.233 ± 0.04	3.755 ± 0.07	-28.95
Fengycin	-6.75 ± 0.04	1.943 ± 0.04	2.4952 ± 0.05	- 24.35
Fusaricidin A	-6.57 ± 0.7	2.117 ± 0.04	6.987 ± 0.07	- 20.61
Fusaricidin B	-6.32 ± 0.04	21.597 ± 0.34	25.626 ± 0.36	- 21.06
Iturin C	-5.91 ± 0.05	4.593 ± 0.6	11.398 ± 0.08	- 20.78
Iturin D	-6.45 ± 0.06	25.616 ± 0.47	29.890 ± 0.42	- 21.46
Polymyxin B	-5.65 ± 0.05	3.081 ± 0.05	12.161 ± 0.18	- 20.74
Surfactin	-4.97 ± 0.05	1.707 ± 0.05	3.257 ± 0.06	- 20.37

Table S14. Molecular interactions between bacillomycin and the O76082 binding site

Type of Bond	Interaction between	Distance (in Å)
Conventional Hydrogen Bond	A:ARG169:HH21 - Bacillomycin:UNK1:O5	2.76007
Conventional Hydrogen Bond	A:ARG169:HH22 - Bacillomycin:UNK1:O5	2.61348
Conventional Hydrogen Bond	A:ARG227:HH11 - Bacillomycin:UNK1:O13	2.21081
Conventional Hydrogen Bond	A:ARG227:HH22 - Bacillomycin:UNK1:O13	2.33934
Conventional Hydrogen Bond	A:ARG282:HH11 - Bacillomycin:UNK1:O13	2.83422
Conventional Hydrogen Bond	A:ARG282:HH12 - Bacillomycin:UNK1:O13	2.91478

https://doi.org/10.3390/biotech15010004

Conventional Hydrogen Bond	Bacillomycin:UNK1:H36 - A:ASP165:OD2	2.03457
Conventional Hydrogen Bond	Bacillomycin:UNK1:H45 - A:SER157:O	2.39836
Conventional Hydrogen Bond	Bacillomycin:UNK1:H48 - A:ASP165:OD2	2.70724
Pi-Alkyl	A:PHE158 - Bacillomycin:UNK1	4.28507
Pi-Alkyl	Bacillomycin:UNK1 - A:ALA451	5.29439

Table S15. Molecular interactions between plipastatin and the O76082 binding site

**Type of Bond**	**Interaction between**	**Distance (in Å)**
Conventional Hydrogen Bond	A:ARG227:HH11 - A:plipastatin:O8	2.25765
Conventional Hydrogen Bond	A:ARG227:HH22 - A:plipastatin:O8	2.78359
Conventional Hydrogen Bond	A:ARG282:HH11 - A:plipastatin:O18	1.99009
Conventional Hydrogen Bond	A:TYR447:HH - A:plipastatin:O1	2.42213
Conventional Hydrogen Bond	A:ASN460:HD22 - A:plipastatin:O11	2.21667
Conventional Hydrogen Bond	A:ARG471:HE - A:plipastatin:O2	1.81225
Conventional Hydrogen Bond	A:ARG471:HH22 - A:plipastatin:O2	2.35075
Conventional Hydrogen Bond	A:plipastatin:H47 - A:SER231:O	2.58792
Conventional Hydrogen Bond	A:plipastatin:HN1 - A:TYR211:OH	2.34558
Conventional Hydrogen Bond	A:plipastatin:H79 - A:ASN460:OD1	2.63889
Conventional Hydrogen Bond	A:plipastatin:H92 - A:GLU220:OE1	2.50353
Carbon Hydrogen Bond	A:ARG282:HD2 - A:plipastatin:O18	2.45673
Carbon Hydrogen Bond	A:ASN460:HA - A:plipastatin:O13	2.5551
Carbon Hydrogen Bond	A:GLY464:HA1 - A:plipastatin:O17	2.37876
Carbon Hydrogen Bond	A:SER467:HB2 - A:plipastatin:O17	2.84345
Carbon Hydrogen Bond	A:plipastatin:C45 - A:ASP165:OD1	3.52615

Pi-Anion	A:ASP3:OD1 - A:plipastatin	4.91946

Table S16. Molecular interactions between fengycin and the O76082 binding site

Type of Bond	Interaction between	Distance (in Å)
Conventional Hydrogen Bond	A:Fengycin:H - A:VAL175:O	2.60865
Carbon Hydrogen Bond	A:GLY182:HA1 - A:Fengycin:O	3.0469
Carbon Hydrogen Bond	A:GLY182:HA2 - A:Fengycin:O	2.93747
Pi-Alkyl	A:PHE167 - A:Fengycin	3.80137
Pi-Alkyl	A:PHE167 - A:Fengycin	4.78523
Pi-Alkyl	A:TRP274 - A:Fengycin	5.47667
Pi-Alkyl	A:Fengycin - A:VAL175	4.90923
Pi-Alkyl	A:Fengycin - A:VAL271	3.92718
Pi-Sigma	A:Fengycin:C - A:TRP274	3.63007

Table S17. Molecular interactions between fusaricidin A and the O76082 binding site

Type of Bond	Interaction between	Distance (in Å)
Conventional Hydrogen Bond	A:Fusaricidin A:H - A:ASP165:OD2	1.94911
Conventional Hydrogen Bond	A:Fusaricidin A:H - A:GLY464:O	2.9241
Conventional Hydrogen Bond	A:Fusaricidin A:H - A:GLY464:O	2.81849
Conventional Hydrogen Bond	A:Fusaricidin A:H - A:SER157:O	3.0896
Carbon Hydrogen Bond	A:GLY161:HA2 - A:Fusaricidin A:O	2.9618
Alkyl	A:Fusaricidin A - A:MET461	4.87554
Alkyl	A:Fusaricidin A - A:ILE331	5.11874
Alkyl	A:Fusaricidin A - A:MET461	5.23789

2. Molecular dynamics simulations

Table S18. Detailed parameters for the 200-ns molecular dynamics simulations of the NOX4 (Q9NPH5)–lipopeptide complexes.

Simulated Complex (Ligand–Receptor)	Box Size (Å³)*	Number of Atoms	Force Field & Water Model	Treatment of Protonation States	Salt Concen-tration	Number of Replicates
Iturin D–Q9NPH5	93.18 x 156.55 x 71.88	97,672	OPLS4 / TIP3P	Assigned using the Protein Preparation Wizard (Schrödinger Suite) at pH 7.0 ± 2.0	0.15 M NaCl	1
Iturin C–Q9NPH5	93.18 x 156.55 x 71.88	97,722	OPLS4 / TIP3P	Assigned using the Protein Preparation Wizard (Schrödinger Suite) at pH 7.0 ± 2.0	0.15 M NaCl	1
Plipastatin – Q9NPH5	93.18 x 156.55 x 71.88	97,764	OPLS4 / TIP3P	Assigned using the Protein Preparation Wizard (Schrödinger Suite) at pH 7.0 ± 2.0	0.15 M NaCl	1

*The orthorhombic solvation box was built using the Desmond System Builder with a 10-Å buffer from the protein surface to the box edge in all directions. The systems were neutralized with Na^+ counterions. Three independent production runs were performed for each complex, initiated with different random velocity seeds.

2.1. Profile of per-residue interaction occupancies for the Q9NPH5 complexes.

Using Panels (a), (b), and (c) represent the complexes with iturin D, plipastatin, and iturin C, respectively. The histograms quantify the fraction of simulation time (% contact) during which specific protein residues engage in interactions with the ligand, categorized into four distinct types: hydrogen bonds (H-bonds, blue), hydrophobic contacts (green), ionic interactions (red), and water bridges (cyan). Interaction criteria follow the standard geometric definitions of the Desmond molecular dynamics suite. Residues exhibiting high occupancy values (>50-70%) constitute the primary interaction network responsible for stabilizing the ligand within the binding site. This quantitative analysis directly corroborates and extends the key interactions identified in the molecular docking poses.

https://doi.org/10.3390/biotech15010004

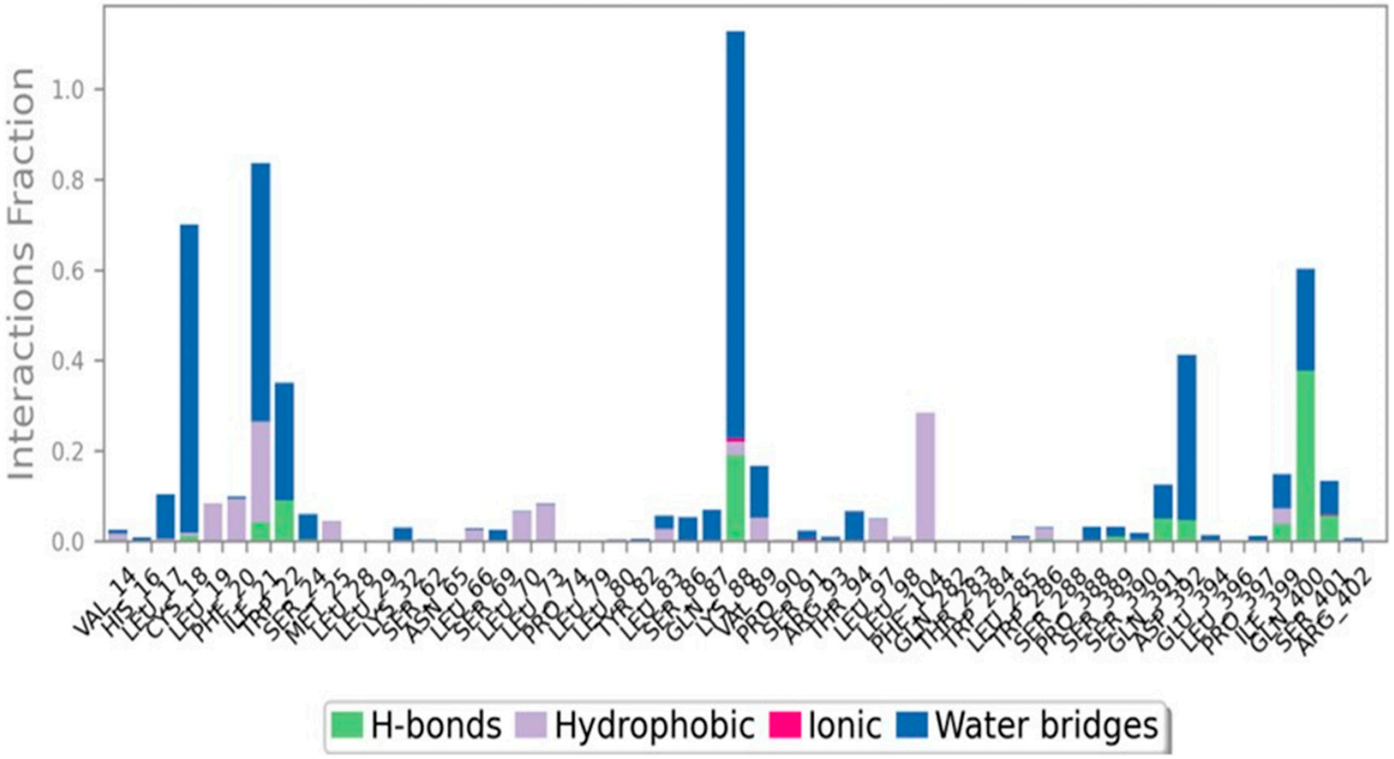
Interactions Fraction
1.0
0.8
0.6
0.4
0.2
0.0
H-bonds
Hydrophobic
Ionic
Water bridges

(**a**)

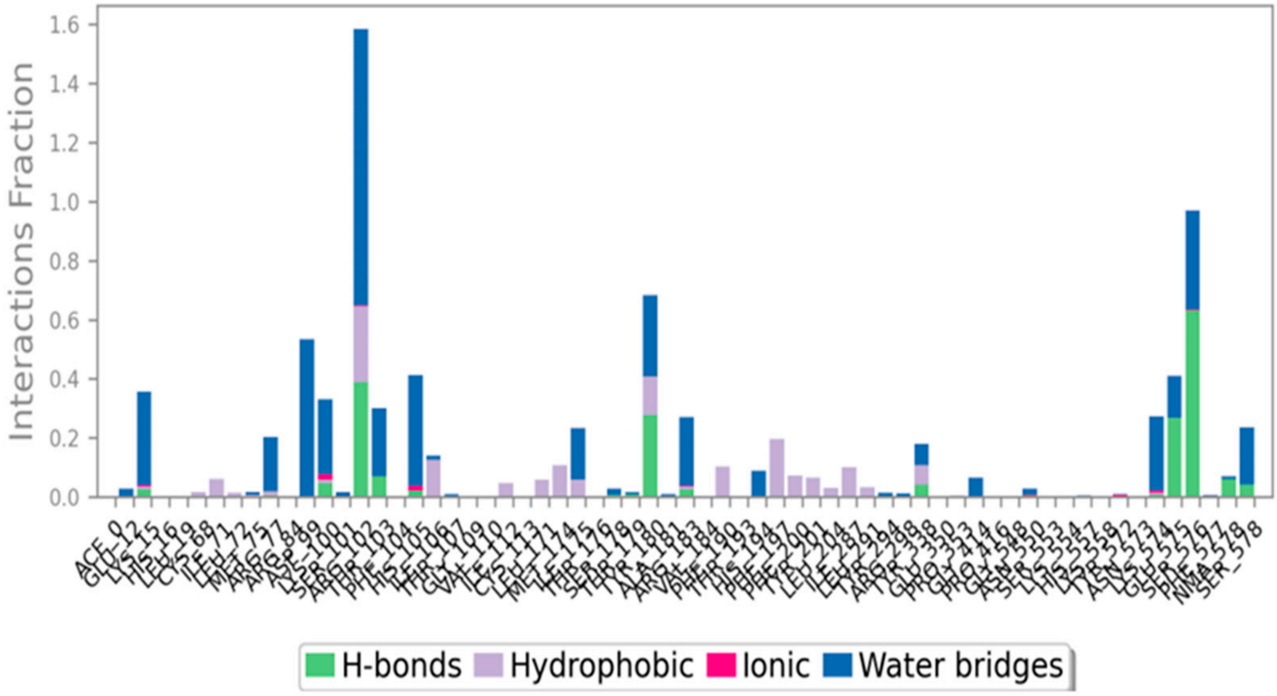
Interactions Fraction
1.6
1.4
1.2
1.0
0.8
0.6
0.4
0.2
0.0
H-bonds
Hydrophobic
Ionic
Water bridges

(**b**)

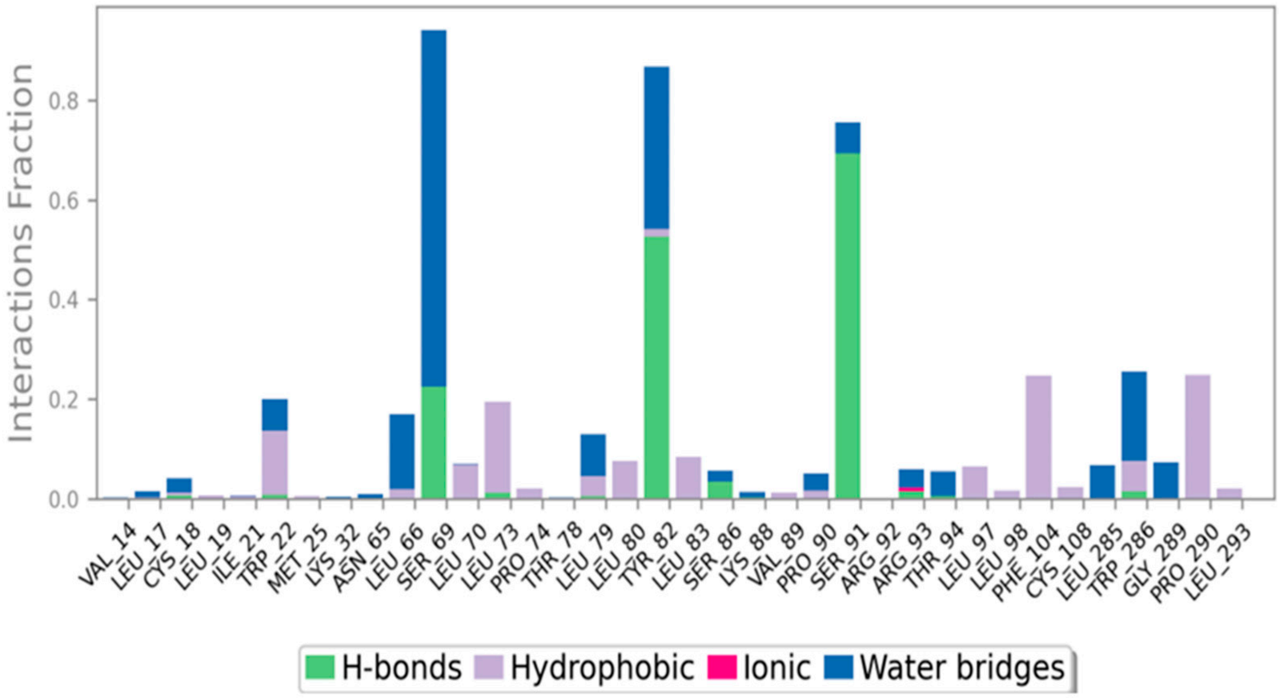

(**c**)

Figure S1. Q9NPH5 interactions with (a) iturin D, (b) plipastatin, and (c) iturin C can be monitored throughout the simulation. Protein-ligand interactions (or contacts) are categorized into four types: Hydrogen Bonds, Hydrophobic, Ionic and Water Bridges.

2.2. Timeline representation of protein-ligand contacts over the 200-ns simulation trajectory.

The 200 nsec molecular dynamics simulations confirmed the stability of the lipopeptide-NOX4 complexes.

The plipastatin-NOX4 complex stabilized after ~50 nsec. The binding was characterized by a high number of persistent contacts, predominantly hydrophobic interactions with residues Phe197, Phe200, His105, and Leu299 (Figure S1a).

The iturin C-NOX4 complex stabilized after ~70 nsec. It engaged a broad set of residues (from Val14 to Leu293) via a mixed binding mode, involving hydrophobic contacts (e.g., Leu79, Tyr82, Leu293) and sustained hydrogen bonding/ionic interactions (e.g., with Arg92, Arg93) (Figure S1b).

The iturin D-NOX4 complex demonstrated the highest stability, reaching equilibrium within ~25 nsec. It maintained intense, persistent contacts with key residues, including Tyr82, Arg304, Glu329, and Arg334, dynamically confirming the stability of hydrogen bonds and π-alkyl interactions identified during docking (Figure S1c).

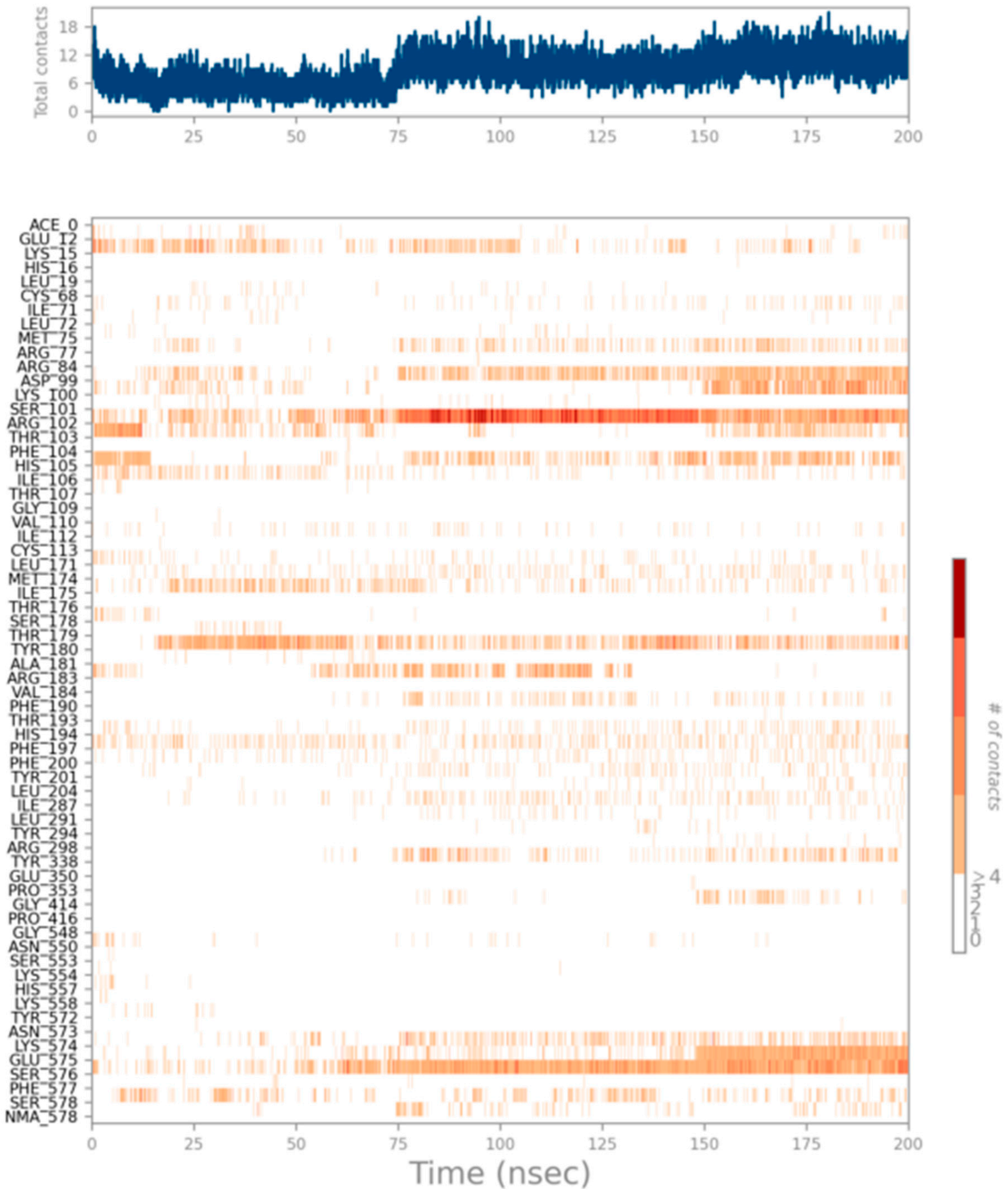
Total contacts
0
6
12
18
0
25
50
75
100
125
150
175
200
ACE_0
GLU_12
LYS_15
HIS_16
LEU_19
CYS_68
ILE_71
LEU_72
MET_75
ARG_77
ARG_84
ASP_99
LYS_100
SER_101
ARG_102
THR_103
PHE_104
HIS_105
ILE_106
THR_107
GLY_109
VAL_110
ILE_112
CYS_113
LEU_171
MET_174
ILE_175
THR_176
SER_178
THR_179
TYR_180
ALA_181
ARG_183
VAL_184
PHE_190
THR_193
HIS_194
PHE_197
PHE_200
TYR_201
LEU_204
ILE_287
LEU_291
TYR_294
ARG_298
TYR_338
GLU_350
PRO_353
GLY_414
PRO_416
GLY_548
ASN_550
SER_553
LYS_554
HIS_557
LYS_558
TYR_572
ASN_573
LYS_574
GLU_575
SER_576
PHE_577
SER_578
NMA_578
Time (nsec)
# of contacts
4
0

(**a**)

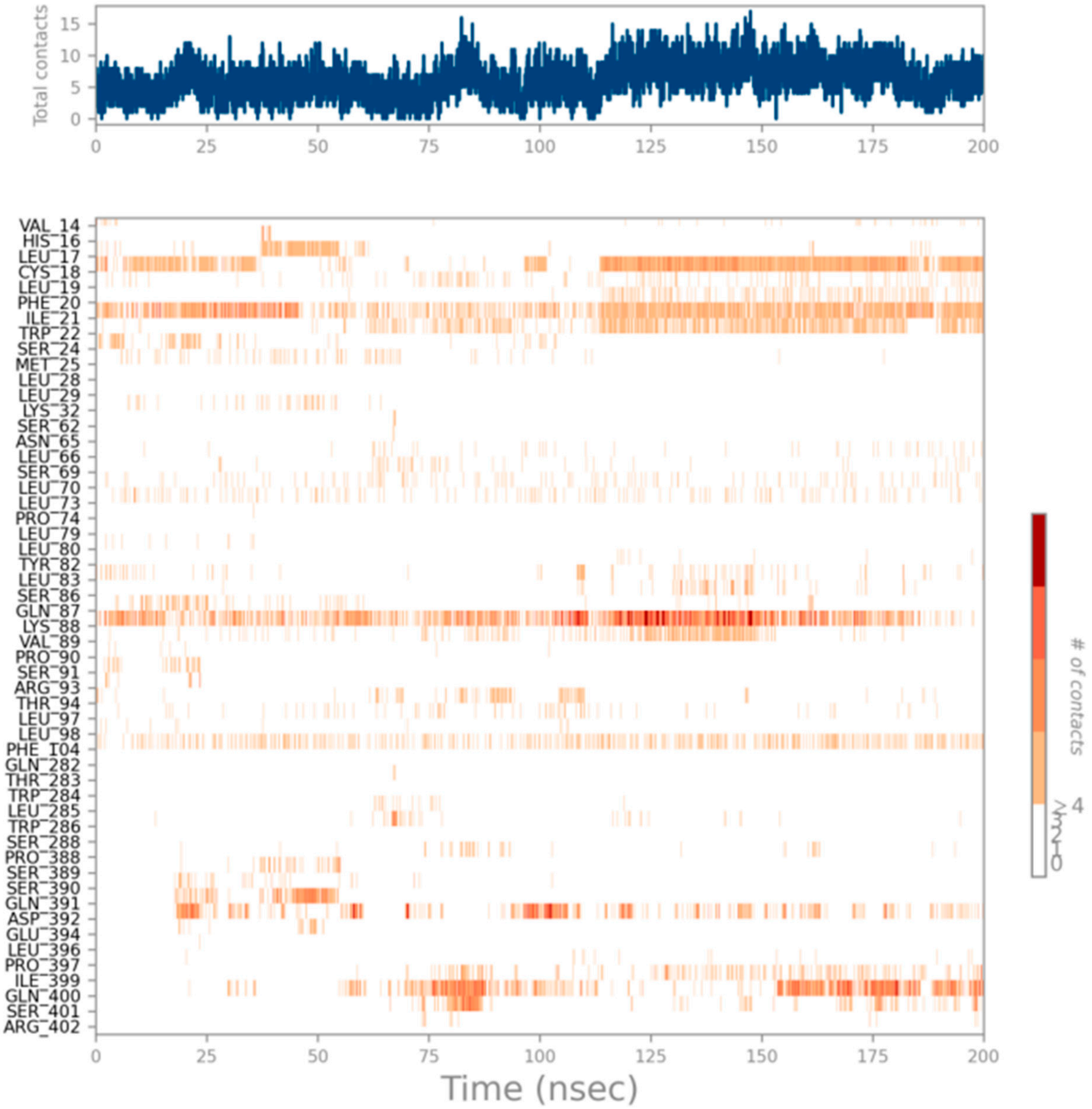
Total contacts
0
5
10
15
0
25
50
75
100
125
150
175
200
VAL_14
HIS_16
LEU_17
CYS_18
LEU_19
PHE_20
ILE_21
TRP_22
SER_24
MET_25
LEU_28
LEU_29
LYS_32
SER_62
ASN_65
LEU_66
SER_69
LEU_70
LEU_73
PRO_74
LEU_79
LEU_80
TYR_82
LEU_83
SER_86
GLN_87
LYS_88
VAL_89
PRO_90
SER_91
ARG_93
THR_94
LEU_97
LEU_98
PHE_104
GLN_282
THR_283
TRP_284
LEU_285
TRP_286
SER_288
PRO_388
SER_389
SER_390
GLN_391
ASP_392
GLU_394
LEU_396
PRO_397
ILE_399
GLN_400
SER_401
ARG_402
Time (nsec)
# of contacts

(**b**)

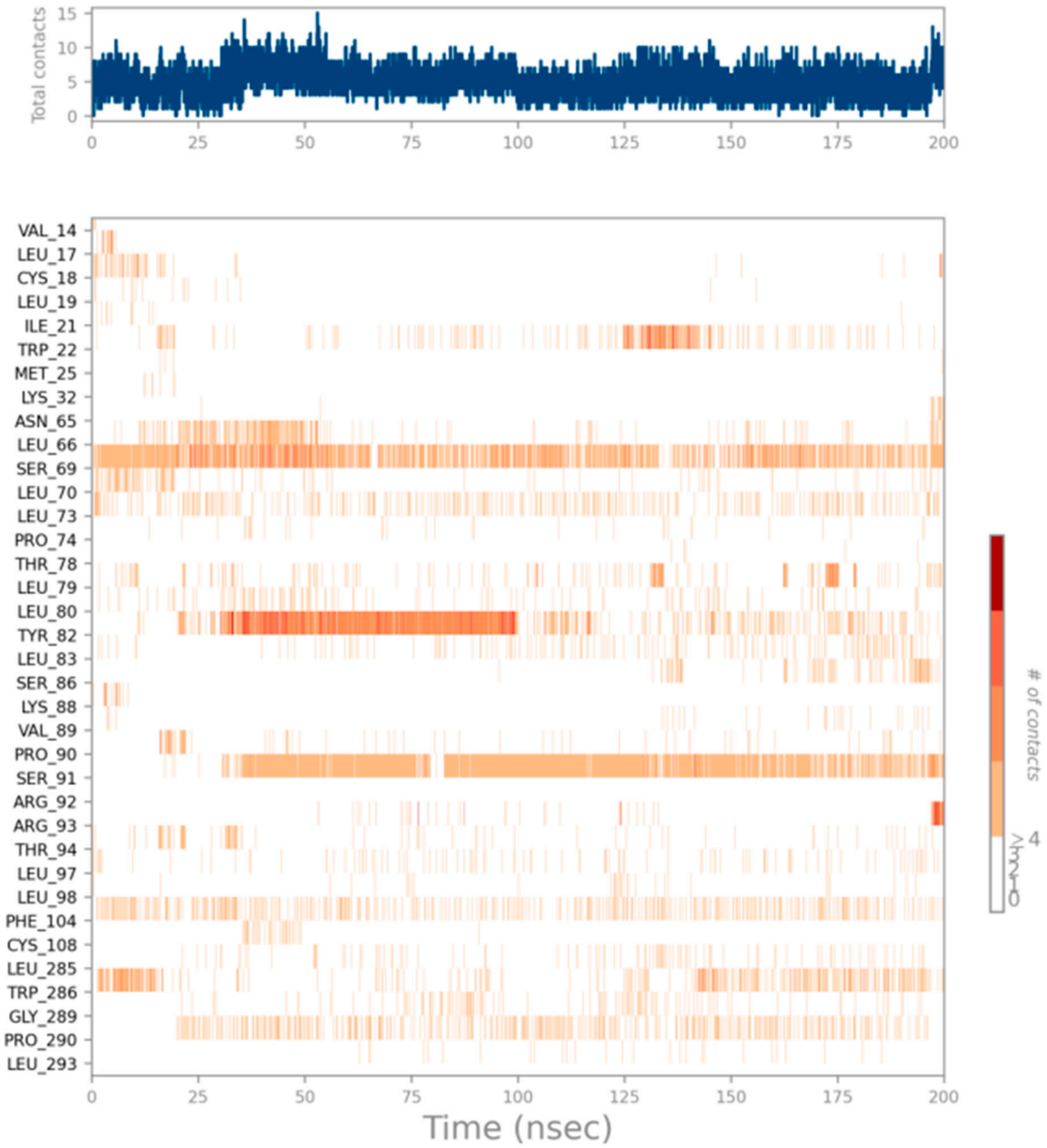

(**c**)

Figure S2. A timeline representation of (a) iturin D–Q9NPH5, (b) plipastatin–Q9NPH5, and (c) iturin C–Q9NPH5 interactions and contacts (H-bonds, Hydrophobic, Ionic, Water bridges).

2.3. Protein backbone flexibility (Cα-RMSF) upon ligand binding.

The root-mean-square fluctuation (RMSF, in Å) of protein Cα atoms is plotted against residue number for the Q9NPH5 complexes with (a) iturin D and (b) iturin C. Peaks indicate regions of high conformational mobility, typically corresponding to solvent-exposed loops. Key residues identified as interacting with the ligand in Figures S1/S2 are marked with vertical green bars. A pronounced suppression of RMSF amplitudes within these ligand-binding regions is observed, demonstrating that ligand binding significantly reduces the intrinsic flexibility of the protein's active site. This damping effect is a hallmark of a stable, well-formed protein-ligand complex.

https://doi.org/10.3390/biotech15010004

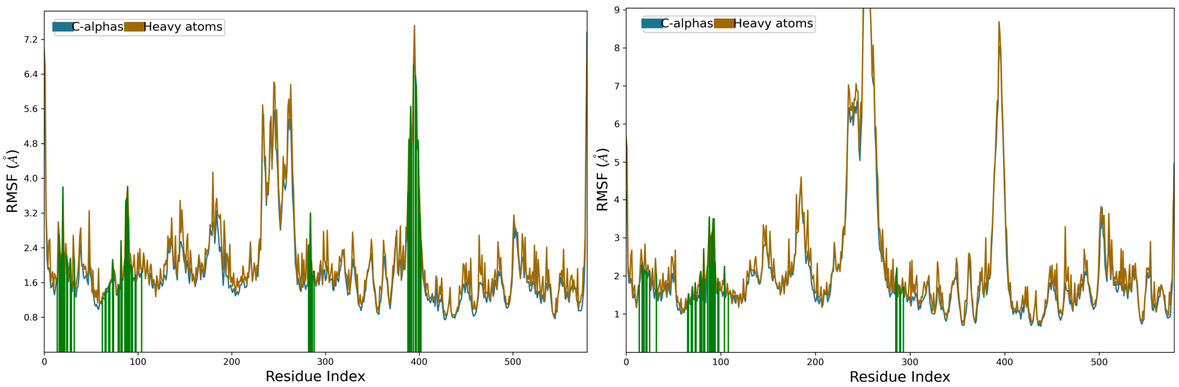

(**a**) (**b**)

Figure S3. Protein RMSF plot for 200 nsec depicting complex stability for (a) iturin D, and (b) iturin C with Q9NPH5. On this plot, peaks indicate areas of the protein that fluctuate the most during the simulation. Protein residues that interact with the ligand are marked with green-colored vertical bars.

2.4. *Conformational flexibility of bound ligands.*

The ligand root-mean-square fluctuation (RMSF, in Å) is decomposed per atom for (a) iturin D and (b) iturin C in complex with Q9NPH5. Each panel juxtaposes the 2D chemical structure of the ligand (top), with atom numbering, against a bar graph of the RMSF for each corresponding heavy atom (bottom). High RMSF values correspond to flexible molecular moieties (e.g., terminal aliphatic chains or unconstrained side groups), while low RMSF values indicate atoms that are rigidly positioned within the binding pocket, often involved in key intermolecular interactions. This atom-level analysis explains the overall ligand RMSD trends by distinguishing between core anchoring points and flexible peripheral regions of the molecules.

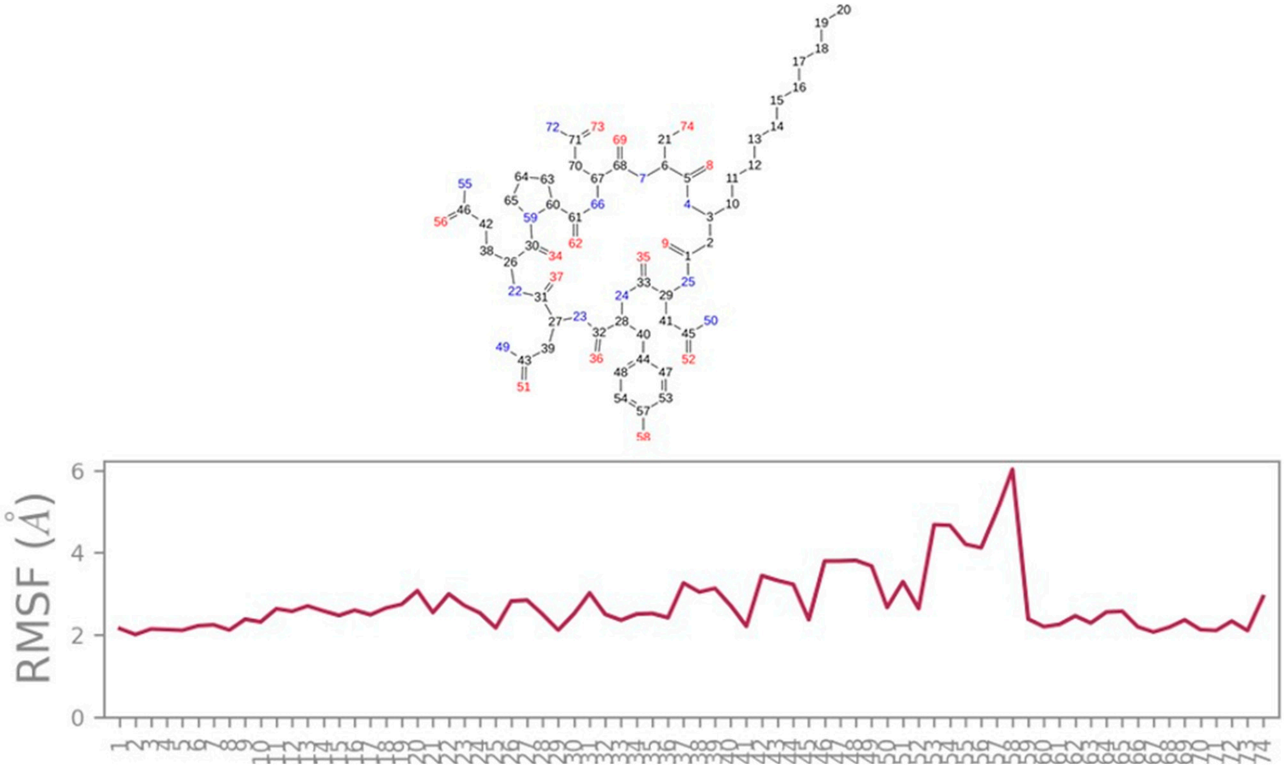

(**a**)

https://doi.org/10.3390/biotech15010004

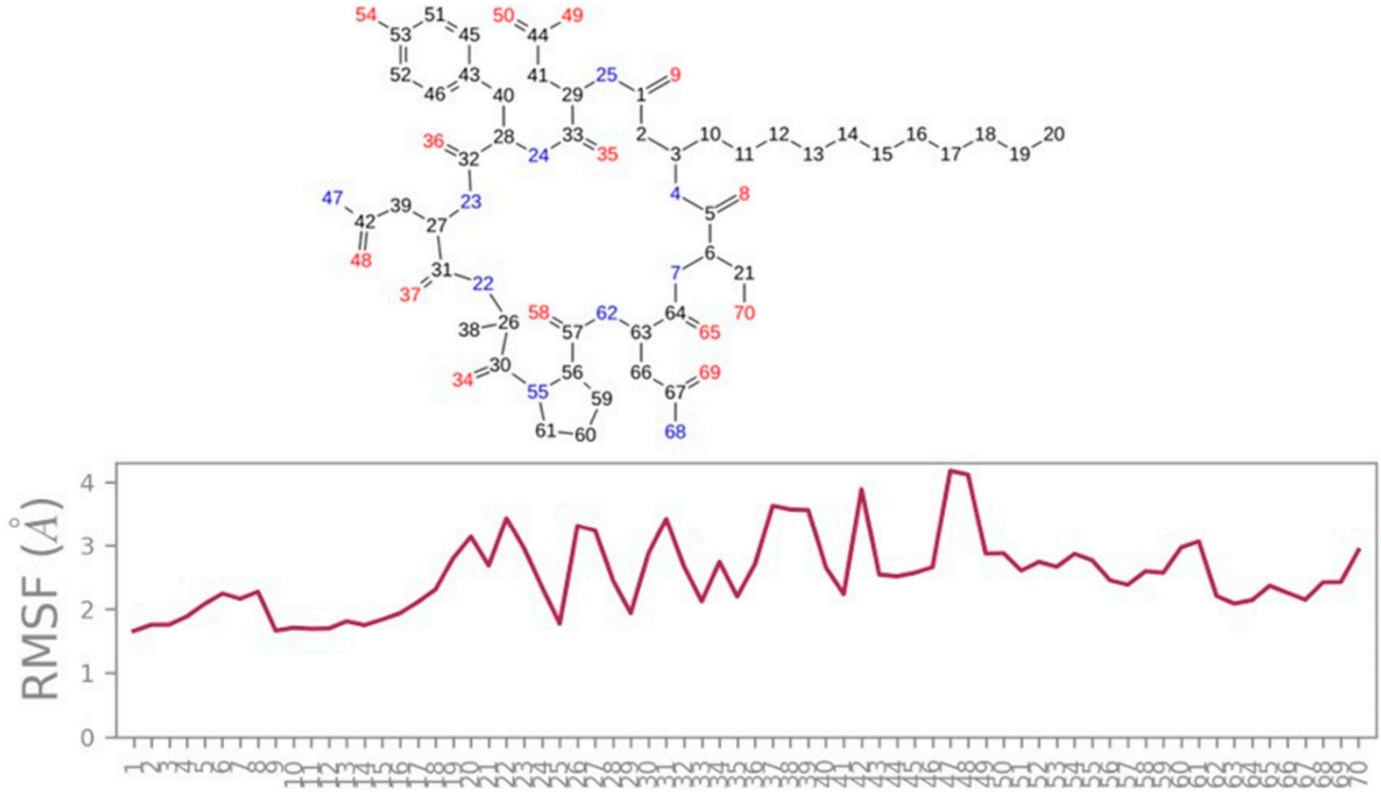

(**b**)

Figure S4. Ligand RMSF plot for 200 nsec depicting complex stability for (a) iturin D, and (b) iturin C with Q9NPH5. Ligand RMSF shows the ligand's fluctuations broken down by atom, corresponding to the 2D structure in the top panel.

3. Re-docking of the top top-ranked ligand with respective receptors, keeping exhaustiveness 32.

Table S19. Configuration used

Complex	Grid-box center	Grid-box size	Exhaustiveness
Iturin D – Q9NPH5	Center X = 10.861<br>Center Y = -5.500<br>Center Z = -3.139	Size X = 84<br>Size Y = 68<br>Size Z = 66	32
Plipastatin –1M17	Center X = 28.592<br>Center Y = -2.43<br>Center Z = 44.665	Size X = 48<br>Size Y = 48<br>Size Z = 62	32
Iturin D – 5GRN	Center X = 18.250<br>Center Y = -11.056<br>Center Z = -11.417	Size X = 30<br>Size Y = 40<br>Size Z = 40	32
Bacillomycin – O76082	Center X = -7.778<br>Center Y = 6.111<br>Center Z = 2.500	Size X = 40<br>Size Y = 40<br>Size Z = 40	32

3.1. Docking of iturin D with NADPH Oxidase 4 (Q9NPH5)

Even after increasing exhaustiveness from 8 to 32, Iturin D retained the same binding orientation in Q9NPH5, preserving key interactions with Arg304, Arg334, Asn330 and Glu329. The docking score showed only a marginal change (−7.86 ± 0.50 → −7.7 kcal/mol), while the RMSD values remained comparably low (1.193 ± 0.05 / 1.153 ± 0.04 Å previously vs. 1.726 / 2.113 Å now), confirming the same binding mode. Therefore, the original

exhaustiveness 8 was already sufficient to capture the correct pose and relevant residue interactions.

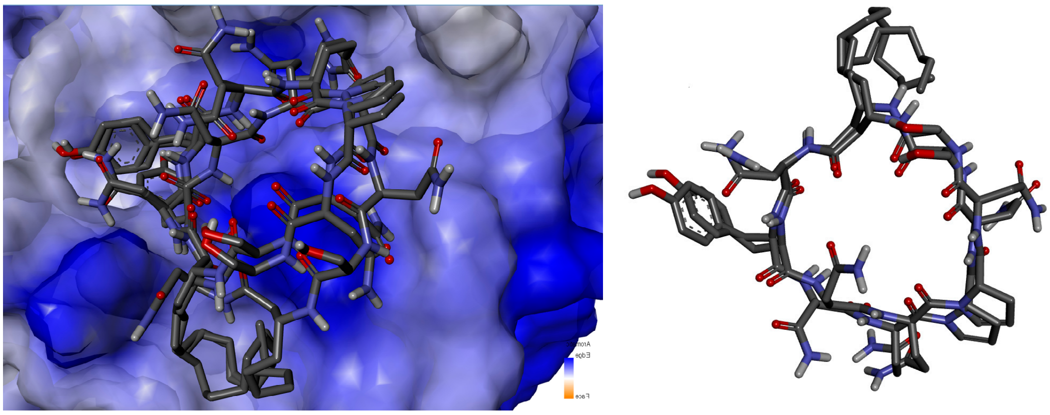

Figure S5. Iturin D docked with Q9NPH5, keeping exhaustiveness 8 and 32, pose aligned Iturin D and Q9NPH5docking with 8 exhaustiveness (left) 32 exhaustiveness (right).

3.2. Docking of plipastatin with EGFR (1M17)

Following the reviewer's recommendation, we performed a redocking of Plipastatin with 1M17, obtaining a binding energy of –10.96 ± 0.5 kcal/mol, with RMSD values of 0.960 Å (LB) and 1.893 Å (UB), confirming good pose convergence in Vina. These values are consistent with our earlier docking results (exhaustiveness = 8), which produced a similar binding energy of –11.12 ± 0.5 kcal/mol, indicating that the ligand's affinity is reproducibly stable across independent simulations. Importantly, the updated interaction profile retains all major binding contacts observed previously, including key residues such as Thr830, Asp831, Asp746, Cys751, Met742, Lys828, Arg817, and Phe832, demonstrating strong methodological consistency.

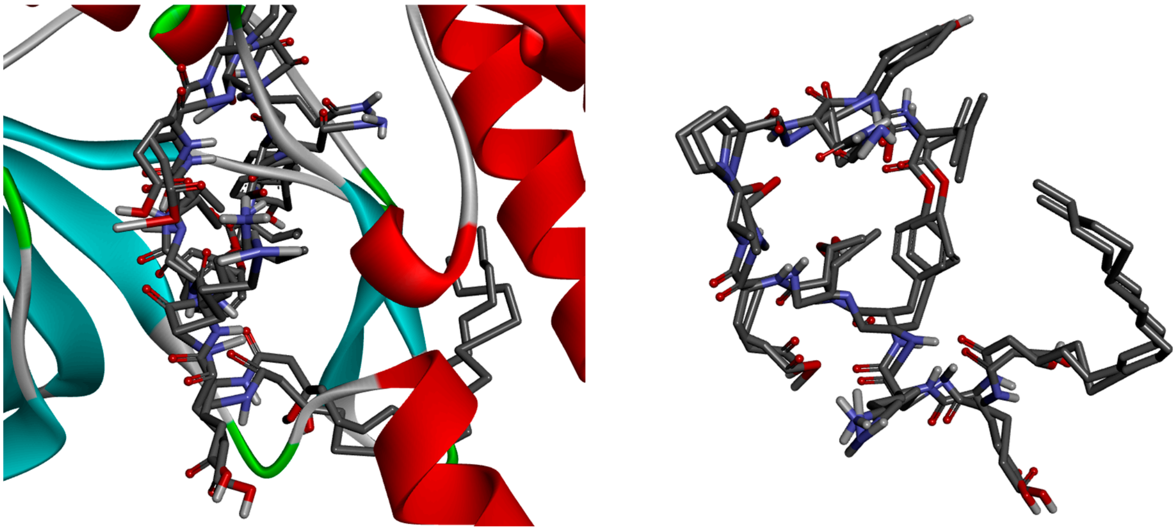

Figure S6. Plipastatin poses with previous docking with exhaustiveness 8 (left) aligned with redocking with exhaustiveness 32 (right).

3.3. Docking of iturin D with PDGFR (5GRN)

After redocking Iturin D with 5GRN using an increased exhaustiveness of 32, the ligand exhibited a binding energy of –8.67 ± 0.6 kcal/mol, with improved pose stability reflected by RMSD values of 1.526 Å (LB) and 2.365 Å (UB). The updated interaction map shows that Iturin D now forms hydrogen bonds with Leu599, Asp846, and Phe678, along with multiple hydrophobic contacts involving Val607, Leu825, Cys835, and Phe837. Notably, Leu599 and Phe837, which were also present in

https://doi.org/10.3390/biotech15010004

the earlier docking run (exhaustiveness 8), remain key anchoring residues, demonstrating strong reproducibility across independent simulations. Although minor variations in contact residues occurred, the overall interaction pattern remains consistent, reaffirming the stability of the predicted binding mode.

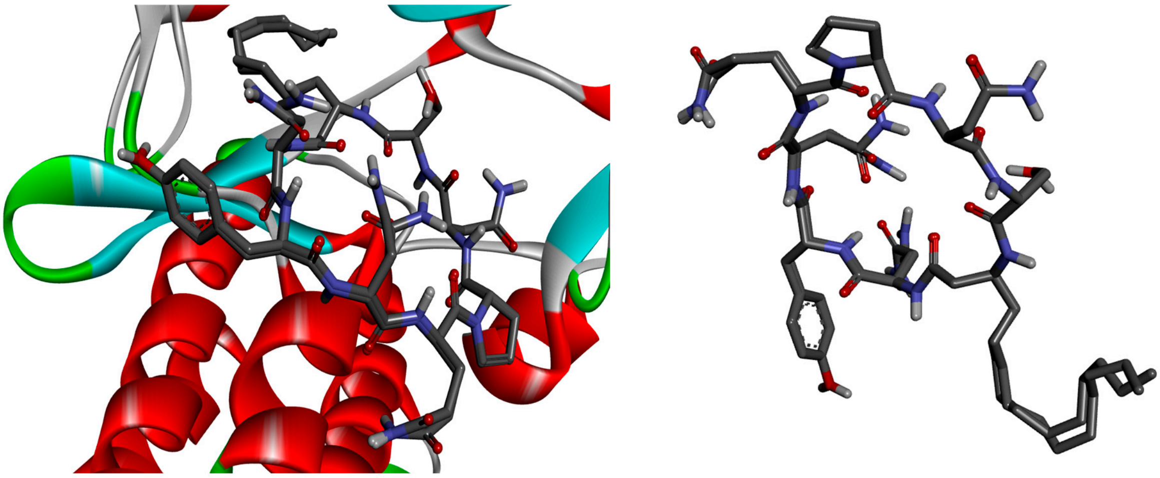

Figure S7. Docked poses with previous docking with exhaustiveness 8 (left) aligned with redocking with exhaustiveness 32 (right), both poses aligned in the binding cavity of the PDGFR.

3.4. Docking of bacillomycin with OCTN2 (O76082)

To validate the stability of Bacillomycin– O76082 binding, the docking was repeated with an increased exhaustiveness of 32, resulting in a binding energy of –9.63 ± 0.05 kcal/mol, which is nearly identical to the previous value of –9.66 ± 0.05 kcal/mol obtained at exhaustiveness 8. The refined docking produced RMSD values of 2.141 ± 0.06 Å (LB) and 2.593 ± 0.05 Å (UB), showing slightly improved pose convergence relative to the earlier run. The updated interaction profile reveals persistent hydrogen bonds with Arg227, Asn460, and Asp165, which were also present previously, confirming these residues as conserved stabilizing contacts. Additionally, bacillomycin continues to form hydrophobic π-interactions with residues such as Tyr447, Val448, and Ala451, consistent with the interaction pattern observed earlier.

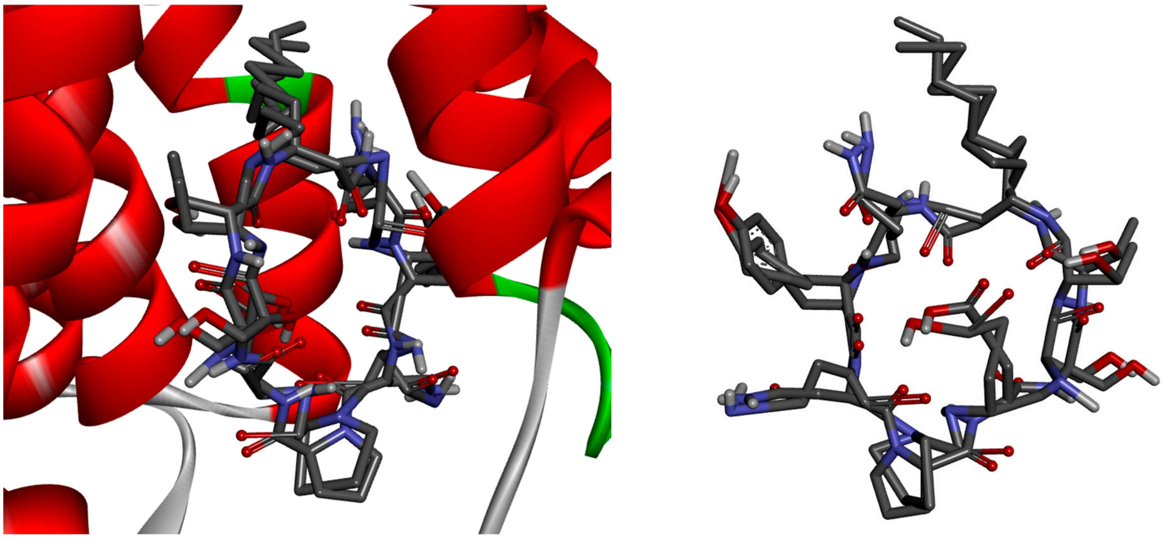

Figure S8. Docked poses of Bacillomycin with OCTN2; previous docking with exhaustiveness 8 (left) aligned with redocking with exhaustiveness 32 (right), both poses aligned in the binding cavity of the PDGFR.

https://doi.org/10.3390/biotech15010004

The redocking clearly validates the docking procedure, as it successfully reproduces the key interaction features observed in the native co-crystal structure of 1M17. The critical Pi-donor hydrogen bond with Thr830:OG1 is maintained, and the dominant Pi-Sigma interactions with LEU694 and LEU820 are fully conserved, showing accurate recovery of essential polar and aromatic contacts. Additionally, the hydrophobic network involving Lys721, Ala719, Met769, and Leu820 is consistently reproduced, confirming that the ligand occupies the same binding cavity with the correct orientation. These conserved interaction fingerprints demonstrate that your docking protocol reliably mimics the native binding mode and is therefore validated.

Table S20. Interaction of co-crystal ligand of 1M17 in native crystal structure

Interaction Between	Types	From	To
A:MET769:N - A:Co crystal Ligands:N2	Conventional Hydrogen Bond	A:MET769:N	A:Co crystal Ligands:N2
A:Co crystal Ligands:C19 - A:GLN767:O	Carbon Hydrogen Bond	A:Co crystal Ligands:C19	A:GLN767:O
A:THR830:OG1 - A:Co crystal Ligands	Pi-Donor Hydrogen Bond	A:THR830:OG1	A:Co crystal Ligands
A:LEU694:CD1 - A:Co crystal Ligands	Pi-Sigma	A:LEU694:CD1	A:Co crystal Ligands
A:LEU820:CD1 - A:Co crystal Ligands	Pi-Sigma	A:LEU820:CD1	A:Co crystal Ligands
A:Co crystal Ligands - A:LYS721	Pi-Alkyl	A:Co crystal Ligands	A:LYS721
A:Co crystal Ligands - A:ALA719	Pi-Alkyl	A:Co crystal Ligands	A:ALA719
A:Co crystal Ligands - A:MET769	Pi-Alkyl	A:Co crystal Ligands	A:MET769
A:Co crystal Ligands - A:ALA719	Pi-Alkyl	A:Co crystal Ligands	A:ALA719
A:Co crystal Ligands - A:LEU820	Pi-Alkyl	A:Co crystal Ligands	A:LEU820

Table S21. Redocking of co-crystal Ligands with 1M17

Interaction Between	Types	From	To
A:THR830:OG1 - A:IM17 Ligand redocking	Pi-Donor Hydrogen Bond	A:THR830:OG1	A:IM17 Ligand redocking
A:LEU694:CD1 - A:IM17 Ligand redocking	Pi-Sigma	A:LEU694:CD1	A:IM17 Ligand redocking
A:LEU820:CD1 - A:IM17 Ligand redocking	Pi-Sigma	A:LEU820:CD1	A:IM17 Ligand redocking

https://doi.org/10.3390/biotech15010004

A:IM17 Ligand re-docking - A:VAL702	Pi-Alkyl	A:IM17 Ligand redocking	A:VAL702
A:IM17 Ligand re-docking - A:LYS721	Pi-Alkyl	A:IM17 Ligand redocking	A:LYS721
A:IM17 Ligand re-docking - A:ALA719	Pi-Alkyl	A:IM17 Ligand redocking	A:ALA719
A:IM17 Ligand re-docking - A:MET769	Pi-Alkyl	A:IM17 Ligand redocking	A:MET769
A:IM17 Ligand re-docking - A:ALA719	Pi-Alkyl	A:IM17 Ligand redocking	A:ALA719
A:IM17 Ligand re-docking - A:LEU820	Pi-Alkyl	A:IM17 Ligand redocking	A:LEU820

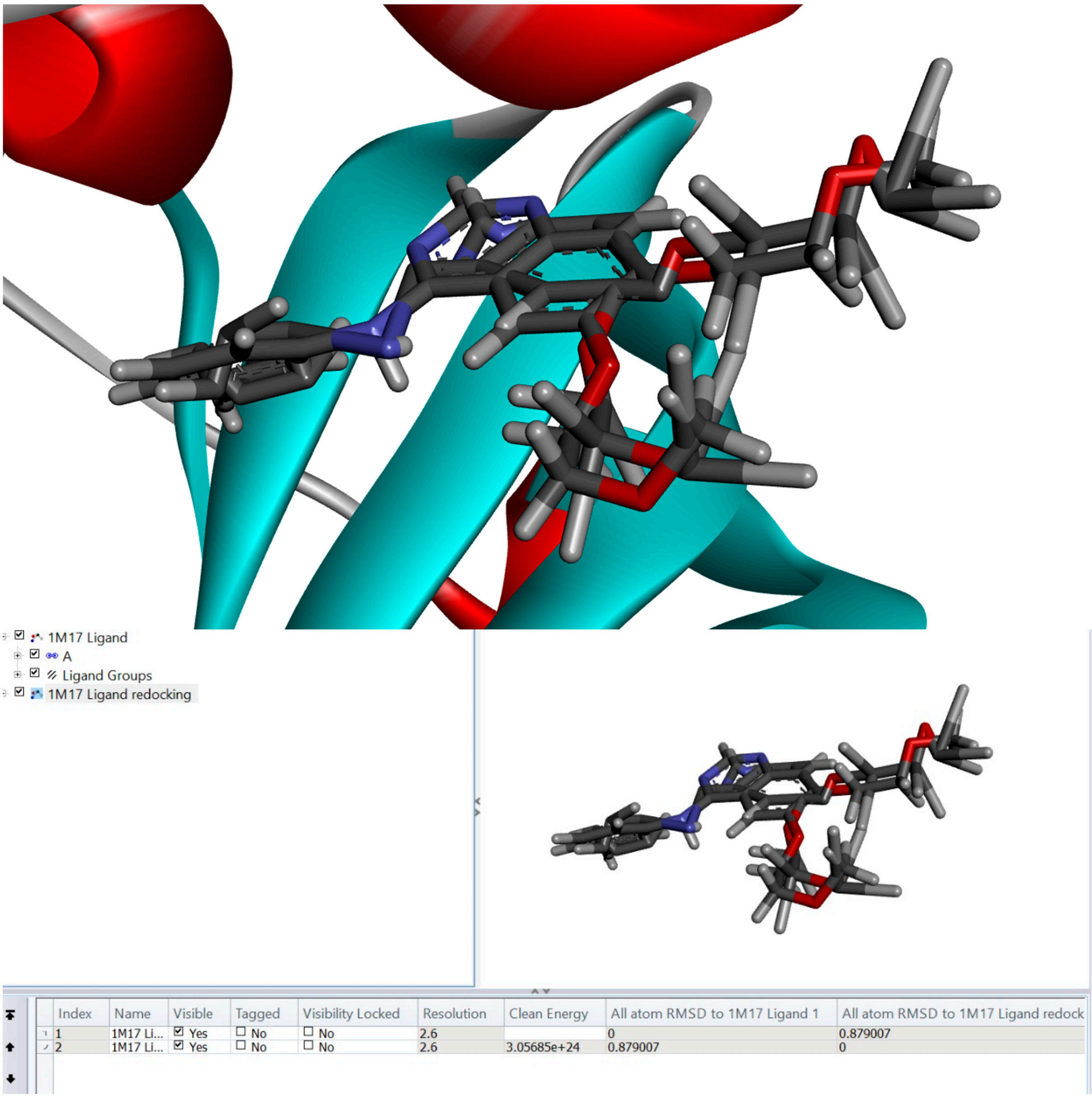

Figure S9. Re-docking of co-crystal ligands, with 1M17.
